# Deep learning‐based whole‐brain B_1_ ^+^‐mapping at 7T

**DOI:** 10.1002/mrm.30359

**Published:** 2024-10-27

**Authors:** Felix Krueger, Christoph Stefan Aigner, Max Lutz, Layla Tabea Riemann, Katja Degenhardt, Kimon Hadjikiriakos, Felix Frederik Zimmermann, Kerstin Hammernik, Jeanette Schulz‐Menger, Tobias Schaeffter, Sebastian Schmitter

**Affiliations:** ^1^ Physikalisch‐Technische Bundesanstalt Berlin Germany; ^2^ Einstein Centre Digital Future Technische Universität Berlin, Biomedical Engineering Berlin Germany; ^3^ Institute for Applied Medical Informatics, Center for Experimental Medicine University Medical Center Hamburg‐Eppendorf Hamburg Germany; ^4^ School of Computation, Information and Technology Technical University of Munich Munich Germany; ^5^ Charité–Universitätsmedizin Berlin, corporate member of Freie Universität Berlin and Humboldt‐Universität Zu Berlin Experimental Clinical Research Center Berlin Germany; ^6^ Working Group On CMR, Experimental and Clinical Research Center, a joint cooperation between the Charité Medical Faculty and the Max‐Delbrueck Center for Molecular Medicine Berlin Germany; ^7^ DZHK (German Centre for Cardiovascular Research), partner site Berlin Berlin Germany; ^8^ Department of Cardiology and Nephrology HELIOS Hospital Berlin‐Buch Berlin Germany; ^9^ Center for Magnetic Resonance Research University of Minnesota Minneapolis Minnesota USA; ^10^ Medical Physics in Radiology German Cancer Research Center (DKFZ) Heidelberg Germany

**Keywords:** 7 tesla, B_1_ ^+^‐mapping, brain, deep learning, parallel transmission

## Abstract

**Purpose:**

This study investigates the feasibility of using complex‐valued neural networks (NNs) to estimate quantitative transmit magnetic RF field (B_1_^+^) maps from multi‐slice localizer scans with different slice orientations in the human head at 7T, aiming to accelerate subject‐specific B_1_^+^‐calibration using parallel transmission (pTx).

**Methods:**

Datasets containing channel‐wise B_1_^+^‐maps and corresponding multi‐slice localizers were acquired in axial, sagittal, and coronal orientation in 15 healthy subjects utilizing an eight‐channel pTx transceiver head coil. Training included five‐fold cross‐validation for four network configurations: ${\mathrm{NN}}_{\mathrm{cx}}^{\mathrm{tra}}$ used transversal, ${\mathrm{NN}}_{\mathrm{cx}}^{\mathrm{sag}}$ sagittal, ${\mathrm{NN}}_{\mathrm{cx}}^{\mathrm{cor}}$ coronal data, and ${\mathrm{NN}}_{\mathrm{cx}}^{\mathrm{all}}$ was trained on all slice orientations. The resulting maps were compared to B_1_^+^‐reference scans using different quality metrics. The proposed network was applied in‐vivo at 7T in two unseen test subjects using dynamic kt‐point pulses.

**Results:**

Predicted B_1_^+^‐maps demonstrated a high similarity with measured B_1_^+^‐maps across multiple orientations. The estimation matched the reference with a mean relative error in the magnitude of (2.70 ± 2.86)% and mean absolute phase difference of (6.70 ± 1.99)° for transversal, (1.82 ± 0.69)% and (4.25 ± 1.62)° for sagittal (${\mathrm{NN}}_{\mathrm{cx}}^{\mathrm{sag}}$), as well as (1.33 ± 0.27)% and (2.66 ± 0.60)° for coronal slices (${\mathrm{NN}}_{\mathrm{cx}}^{\mathrm{cor}}$) considering brain tissue. ${\mathrm{NN}}_{\mathrm{cx}}^{\mathrm{all}}$ trained on all orientations enables a robust prediction of B_1_^+^‐maps across different orientations. Achieving a homogenous excitation over the whole brain for an in‐vivo application displayed the approach's feasibility.

**Conclusion:**

This study demonstrates the feasibility of utilizing complex‐valued NNs to estimate multi‐slice B_1_^+^‐maps in different slice orientations from localizer scans in the human brain at 7T.

## 1 INTRODUCTION

Ultra‐high field (UHF; ≥7T) MRI allows for increased spectral, spatial, and/or temporal resolution and, in many cases, improved contrast compared to lower field strengths.^1^ However, a fundamental problem at UHF is the spatial heterogeneity of the transmit (Tx) magnetic RF field (B_1_^+^) required to excite or manipulate the magnetization, yielding spatially heterogeneous flip‐angles (FAs) and, thus, heterogeneous signals and contrast.^2^, ^3^ One effective solution is parallel transmission (pTx). Here, channel‐independent static or dynamic RF pulses^2^, ^3^, ^4^, ^5^ are used for multi‐Tx‐channel coils. However, even for identical feeding voltages at the coil elements, the resulting B_1_^+^‐field distributions can vary among subjects and even within the same measurement due to respiratory motion.^6^, ^7^, ^8^ Therefore, subject‐tailored pTx pulses are commonly calculated at the beginning of each session based on measured B_1_^+^‐maps. This calibration process is often time‐consuming, requiring up to 15 min,^9^ particularly when focusing on the human body.

Different approaches have been pursued to accelerate this calibration process. Gras et al.^11^ introduced universal pulses: B_1_^+^‐maps are acquired in different subjects before the actual study for a given pTx coil. Based on these datasets, an RF pulse approximating the desired FA pattern across all subjects is calculated offline. Such one‐fits‐all pulses also perform well in unseen subjects and have been applied in various studies in the brain,^10^, ^11^, ^12^ heart,^13^ and spinal cord^14^ at UHF.

Alternatively, rapid B_1_^+^‐mapping procedures,^15^, ^16^, ^17^, ^18^, ^19^, ^20^ such as DREAM^15^ or presaturation‐based B_1_^+^‐mapping,^18^, ^19^, ^20^ can expedite the calibration process. These methods allow channel‐wise acquisitions of 3D or 2D multi‐slice B_1_^+^‐maps of the entire brain within a minute or less. For example, Kent et al.^20^ reduced the acquisition time for 3D B_1_^+^‐maps to under 20 s with eight Tx‐channels utilizing a sandwiched presaturation TurboFLASH sequence.

Both approaches have distinct merits and limitations. Universal pulses eliminate the calibration process within the session, while subject‐tailored techniques typically achieve a better FA homogeneity.^13^, ^16^, ^21^ However, the performance of subject‐specific RF pulses depends on the B_1_^+^‐maps's accuracy. Fast B_1_^+^‐mapping methods often yield less accurate results and/or lower dynamic ranges.^22^, ^23^, ^24^ Furthermore, high acceleration factors may require long reconstruction times, and increasing the resolution or the number of Tx‐channels from eight to 16 or 32^25^, ^26^ extends calibration times to several minutes, even for the human brain.

Recent works applied deep learning (DL) to calibration techniques in UHF MRI,^27^, ^28^, ^29^, ^30^, ^31^, ^32^, ^33^, ^34^, ^35^, ^36^ particularly to acquire B_1_^+^‐fields more rapidly.^34^, ^35^, ^36^ Eberhardt et al.^36^ accelerated channel‐wise B_1_^+^‐mapping for the brain at 7 T, acquiring data for only a fraction of Tx‐channels, while the remaining channels were estimated using DL. Furthermore, a novel DL‐based method for the thorax^37^ approximates relative, complex B_1_^+^‐maps for eight Tx‐channels based on localizers obtained with multiple receive (Rx) channels using a real‐valued neural network (NN). This technique does not add calibration time for B_1_^+^‐mapping, as localizers are acquired by default at the beginning of each session. Yet, in that work, B_1_^+^‐maps were estimated only for transversal 2D slices, and changing the slice orientation requires new training data. Most importantly, although B_1_^+^‐fields are complex‐valued quantities, all introduced DL‐based techniques so far consider solely *real‐valued NNs*. Achieving high performances for complex‐valued data with real‐valued NNs might be plausible, for example, for magnitude image reconstruction.^38^, ^39^ However, for phase‐sensitive applications, such as B_1_^+^‐mapping, *complex‐valued NNs* might enhance the learning process^40^, ^41^ by incorporating mathematically consistent complex operations.^42^, ^43^

This work investigates the benefits of using *complex‐valued* NNs to estimate channel‐wise multi‐slice B_1_^+^‐maps from single localizer images obtained in a CP+ ‐transmission mode for different orientations in the human brain at 7T using an eight‐channel pTx transceiver head‐coil. The proposed *complex‐valued* NNs are compared to corresponding real‐valued architectures and the recently presented DL‐based approach.^37^ The impact of the localizer's orientation on the resulting B_1_^+^‐maps is analyzed, and the performance of four different NNs trained on transversal, sagittal, and coronal slices, as well as on a combined dataset containing all orientations, is evaluated. The accuracy of the predicted (PR) B_1_^+^‐maps is investigated concerning acquired B_1_^+^‐reference scans as ground truth (GT). A subject‐specific calibration pipeline using dynamic 3D kt‐point pTx pulses is investigated by Bloch simulations, and an in‐vivo application is demonstrated.

## 2 METHODS

A custom‐built 8Tx/8Rx‐channel transceiver head‐coil^44^ is used to acquire CP+ ‐like localizers as input to the NNs (c.f. Figure S1), and absolute, Tx‐channel‐wise, multi‐slice B_1_^+^‐maps^45^ (c.f. Supporting Information S1) as unbiased B_1_^+^‐reference (46) in different orientations. Subject‐specific static^37^, ^47^ and dynamic pTx pulses^48^ based on the estimated whole‐brain B_1_^+^‐maps are designed (c.f. Supporting Information S2) to assess the impact of the PR quality on the pulse design. The approach's feasibility is demonstrated in‐vivo (c.f. Supporting Information S3).

### 2.1 NNs and cross‐validation

All NNs transform complex‐valued, Rx‐channel‐wise localizers and the sum‐of‐squares localizer magnitude as the complex input into Tx‐channel‐wise B_1_^+^‐maps for the complex output (c.f. Figure S1). Using complex‐valued NNs preserves the correlation between real and imaginary parts of complex‐valued quantities for both linear (e.g., multiplication) and non‐linear operations (activation function).^41^ Complex convolutions, activations, loss functions, and common network layers, for example, pooling, are redefined for the complex plane.^41^, ^42^, ^43^, ^49^ The architecture of the complex‐valued NNs (termed ${\mathrm{NN}}_{\mathrm{cx}}$) is derived from the recently presented NN (termed ${\mathrm{NN}}_{\text{real}}^{\text{orig}}$) for the thorax^37^ and implemented in Python 3.8.5 using Tensorflow 2.2.0^50^ trained on a 24GB NVIDIA Titan RTX.

The eight Rx‐channels of the complex‐valued localizers and one corresponding sum‐of‐squares magnitude image lead to an input size of 128×96×9. The output size of 128×96×8 is based on the B_1_^+^‐maps for eight Tx‐channels. Multi‐slice input and output with whole‐brain coverage are processed slice‐wise, generating 2D sets of B_1_^+^‐maps for eight Tx‐channels for each 2D localizer input to the NN. ${\mathrm{NN}}_{\mathrm{cx}}$ contains four encoding and decoding stages, each with two sequential (3×3) complex convolutions with (1,1) strides for feature extraction and an additional (3×3) (up‐) convolution using (2,2) strides for up‐ or down‐sampling. A modified rectified linear unit (ModReLU)^43^, ^51^ with Glorot‐uniform‐initialization^52^ is utilized as the complex non‐linear activation function:

(1) $$\text{ModReLU}\left(z\right)=\text{ReLU}\left(\left|z\right|+b\right)e^{i\cdot ∠z}.$$

Here, z represents any arbitrary complex number and b a real‐valued, learnable bias parameter to shift the magnitude with respect to the origin. The number of feature maps is 16 in the first and last encoding/decoding stages and in/decreased two‐fold per stage. Skip connections and dropout layers^49^ are incorporated at the end of each stage. Unlike the original model ${\mathrm{NN}}_{\text{real}}^{\text{orig}}$, the architectures of ${\mathrm{NN}}_{\mathrm{cx}}$ use convolutions for downsampling aiming for improved feature extraction and additional head layers in each Tx‐channel for adaption, consisting of four convolutions with decreasing feature maps.

The complex‐valued encoder‐decoder models proposed in this study are trained by minimizing an unbiased ⊥ + L2 loss.^53^ An adaptive moment estimation optimizer^54^ is used for optimization, with a learning rate starting at 10^−4^, decaying by 0.19% per epoch. Training includes a batch size of 1 for 1000 epochs. The source code and an exemplary dataset are provided: https://github.com/felixkrueger90/ComplexB1.

Four complex NNs are trained with different slice orientations to investigate the impact of the localizer's orientation on the resulting B_1_^+^‐maps. ${\mathrm{NN}}_{\mathrm{cx}}^{\mathrm{tra}}$ is trained on transversal data (164 slices), ${\mathrm{NN}}_{\mathrm{cx}}^{\mathrm{sag}}$ on sagittal (184 slices), and ${\mathrm{NN}}_{\mathrm{cx}}^{\mathrm{cor}}$ on coronal data (222 slices) from 15 subjects. ${\mathrm{NN}}_{\mathrm{cx}}^{\mathrm{all}}$ is trained on a combined library of all orientations (570 slices). A five‐fold split ratio and cross‐validation for all four NNs evaluate the approach's generalization capability. Datasets of 15 subjects containing transversal, sagittal, and coronal Rx‐channel‐wise localizers and corresponding Tx‐channel‐wise B_1_^+^‐maps are randomly divided into five subsets, each containing data with all orientations from three subjects. Each fold uses one subset as test data and the other four for training, repeated five times for all NN configurations (${\mathrm{NN}}_{\mathrm{cx}}^{\mathrm{tra}}$ to ${\mathrm{NN}}_{\mathrm{cx}}^{\mathrm{all}}$) with consistent subject partitioning. ${\mathrm{NN}}_{\mathrm{cx}}^{\mathrm{tra}}$ is compared to corresponding real‐valued networks with increasing numbers of trainable parameters (${\mathrm{NN}}_{\text{real}}^{\mathrm{tra},1}$: 7.8 M, ${\mathrm{NN}}_{\text{real}}^{\mathrm{tra},2}$: 15.9 M, ${\mathrm{NN}}_{\text{real}}^{\mathrm{tra},3}$: 30.5 M) (c.f. Figure S2A), the architecture for the thorax (${\mathrm{NN}}_{\text{real}}^{\text{orig}}$), and a modified version (${\mathrm{NN}}_{\text{real}}^{\mathrm{mod}}$) for the brain (c.f. Figure S2B) to examine the impact of the complex‐valued architecture. The data procession and optimization parameters for the real‐valued NNs can be found in.^37^

Analysis of the quality of the PR (B_1_^+^_PR_) compared to the reference (B_1_^+^_GT_) involves the absolute differences of the complex‐valued quantities B_1_^+^_PR_ and B_1_^+^_GT_ considered for the magnitude $∆\left|B_{1}^{+}\right|=\left|B_{1\;\mathrm{PR}}^{+}-B_{1\;\mathrm{GT}}^{+}\right|$ and phase $∠{∆B}_{1}^{+}=∠\left(B_{1\;\mathrm{PR}}^{+}/B_{1\;\mathrm{GT}}^{+}\right)$, root‐mean‐squared error (RMSE) of the magnitude and complex quantities, and structural similarity index measure (SSIM) for the magnitude, phase, and complex values.

## 3 RESULTS

### 3.1 Network architecture and image quality

**FIGURE 1:**
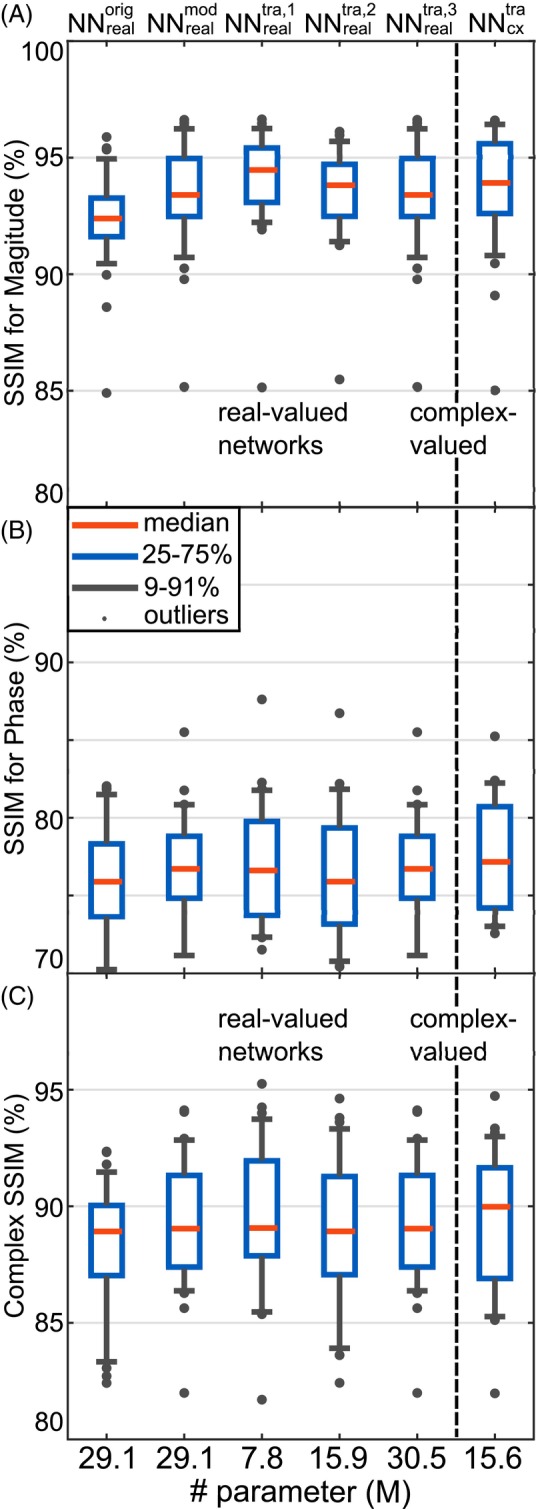
Comparison of the prediction quality assessed by the structure similarity index measure (SSIM) for the magnitude (A), phase (B), and the complex quantity (C) between the complex‐valued network ${\mathrm{NN}}_{\mathrm{cx}}^{\mathrm{tra}}$, corresponding real‐valued networks (${\mathrm{NN}}_{\text{real}}^{\mathrm{tra},1}$ to ${\mathrm{NN}}_{\text{real}}^{\mathrm{tra},3}$), the previously introduced network for the human thorax (${\mathrm{NN}}_{\text{real}}^{\text{orig}}$), and a modified version for the introduced use case (${\mathrm{NN}}_{\text{real}}^{\mathrm{mod}}$). The networks were tested for 32 transversal slices from three unseen subjects. Note the different axis intervals for the phase.

Figure 1 compares the prediction performance assessed by the SSIM for the magnitude (Figure 1A), phase (Figure 1B), and the complex quantity (Figure 1C) of the complex‐valued network ${\mathrm{NN}}_{\mathrm{cx}}^{\mathrm{tra}}$, corresponding real‐valued networks (${\mathrm{NN}}_{\text{real}}^{\mathrm{tra},1}$ to ${\mathrm{NN}}_{\text{real}}^{\mathrm{tra},3}$) with an increasing number of trainable parameters, the network for the thorax (${\mathrm{NN}}_{\text{real}}^{\text{orig}}$), and the modified version for the brain (${\mathrm{NN}}_{\text{real}}^{\mathrm{mod}}$) for 32 unseen transversal slices. In general, the quality metrics indicated an inferior prediction quality for the architecture ${\mathrm{NN}}_{\text{real}}^{\text{orig}}$ constructed for the thorax. The results improved when going to real‐valued NNs, but the number of trainable parameters did not notably influence the results. Furthermore, the prediction quality did not significantly improve for the complex‐valued ${\mathrm{NN}}_{\mathrm{cx}}^{\mathrm{tra}}$ compared to the best real‐valued network ${\mathrm{NN}}_{\text{real}}^{\mathrm{tra},1}$. Furthermore, an evaluation regarding the quantitative agreement (Figure S4) revealed a similar prediction quality for the magnitudes but a trend toward a better prediction quality for the phase for the complex‐valued ${\mathrm{NN}}_{\mathrm{cx}}^{\mathrm{tra}}$.

**FIGURE 2:**
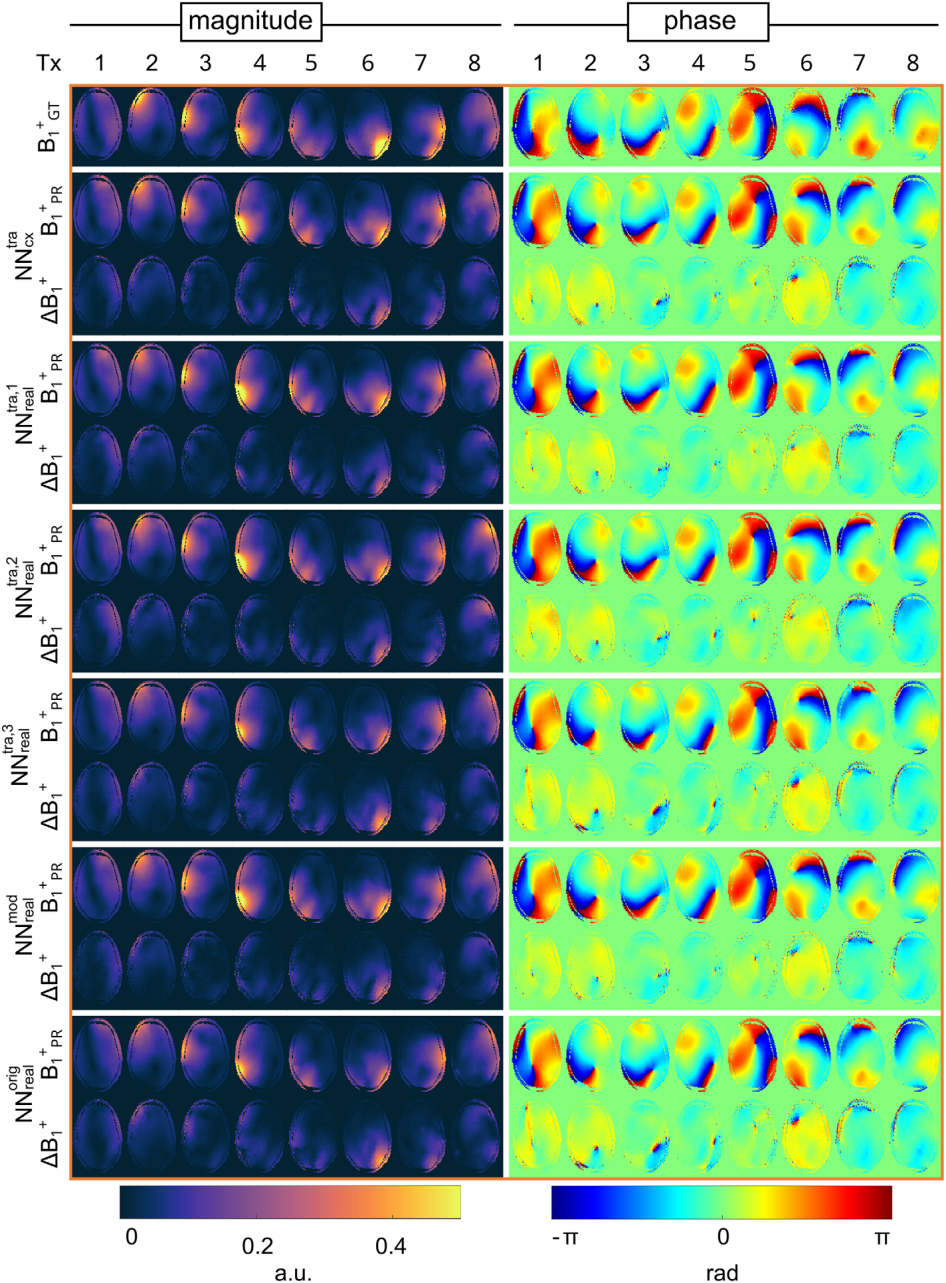
Channel‐wise B_1_^+^‐maps considering the magnitude and phase for the prediction B_1_^+^_PR_ of the complex‐valued network ${\mathrm{NN}}_{\mathrm{cx}}^{\mathrm{tra}}$, corresponding real‐valued networks (${\mathrm{NN}}_{\text{real}}^{\mathrm{tra},1}$ to ${\mathrm{NN}}_{\text{real}}^{\mathrm{tra},3}$) with an increasing number of real‐valued parameters, the previously introduced network for the human thorax (${\mathrm{NN}}_{\text{real}}^{\text{orig}}$) and a modified version for the introduced use case (${\mathrm{NN}}_{\text{real}}^{\mathrm{mod}}$). The results are compared to the B_1_^+^‐reference B_1_^+^_GT_, displayed by the absolute error ΔB_1_^+^ of the magnitude Δ|B_1_^+^| = | B_1_^+^_PR_ − B_1_^+^_GT_ | and phase ∠B_1_^+^ = ∠(B_1_^+^_PR_ / B_1_^+^_GT_) for one exemplary transversal slice of one unseen subject.

Figure 2 shows the channel‐wise B_1_^+^‐maps for one slice, comparing the PR of ${\mathrm{NN}}_{\mathrm{cx}}^{\mathrm{tra}}$ to real‐valued networks ${\mathrm{NN}}_{\text{real}}^{\mathrm{tra},1}$ to ${\mathrm{NN}}_{\text{real}}^{\mathrm{tra},3}$, ${\mathrm{NN}}_{\text{real}}^{\text{orig}}$, and ${\mathrm{NN}}_{\text{real}}^{\mathrm{mod}}$ with the GT. The PR data of all NN matches the GT data, not only for the magnitude but particularly in the phase distributions. Yet, phase differences in regions of low magnitudes, c.f. Tx‐channels 1, 3, and 6 and deviations in the magnitude at high levels between PR and GT are evident.

Further assessment of ${\mathrm{NN}}_{\mathrm{cx}}^{\mathrm{tra}}$ to ${\mathrm{NN}}_{\mathrm{cx}}^{\mathrm{all}}$ regarding the five‐fold cross‐validation (Tables S1–S4) and prediction quality (Figures S5–S11) is provided.

### 3.2 Effect of B_1_ ^+^‐quality on RF pulse design for dynamic pTx

**FIGURE 3:**
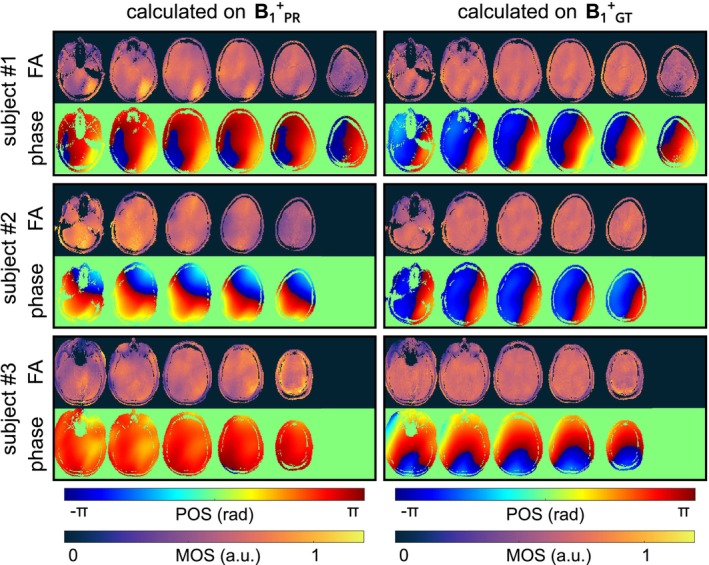
FA and phase prediction of dynamic four kt‐point pulses designed based on the predicted multi‐slice B_1_^+^‐maps (B_1_^+^_PR_) using ${\mathrm{NN}}_{\mathrm{cx}}^{\mathrm{tra}}$ and the B_1_^+^‐reference (B_1_^+^_GT_) data for every second slice of all three unseen datasets of subset #2. Using Bloch simulation, the calculated pulses are applied to the B_1_^+^_GT_ data. The dynamic pulses produce smooth FA distributions over the whole‐brain volume for all unseen subjects. Note the varying numbers of slices due to different head sizes.

Figure 3 illustrates the FA results of four kt‐point pulses optimized based on PR maps of ${\mathrm{NN}}_{\mathrm{cx}}^{\mathrm{tra}}$ and B_1_^+^‐reference data to improve the FA homogeneity for three unseen test subjects. The dynamic pTx pulses improve the homogeneity of the excitation profile and produce smooth FA distributions over multiple slices when applied to the GT. Residual, low‐frequency variation can be seen in lower and upper slices, where the coil has less transmit sensitivity. The CV values calculated over all slices for the three test subjects improved from 27.4%, 28.7%, and 25.1% for the CP+ ‐like mode of the GT data to 13.7%, 16.3%, and 9.7% using four kt‐point pulses calculated on the PR maps of ${\mathrm{NN}}_{\mathrm{cx}}^{\mathrm{tra}}$, indicating a low FA heterogeneity. In comparison, when relying on the acquired B_1_^+^‐reference data for the optimization process, the CV values reduce to 8.2%, 8.9%, and 7.1%. The effect of the B_1_^+^‐quality of the PR data on RF pulse design for static pTx is provided (Supporting Information S5).

### 3.3 Predicting B_1_ ^+^ for different slice orientations

**FIGURE 4:**
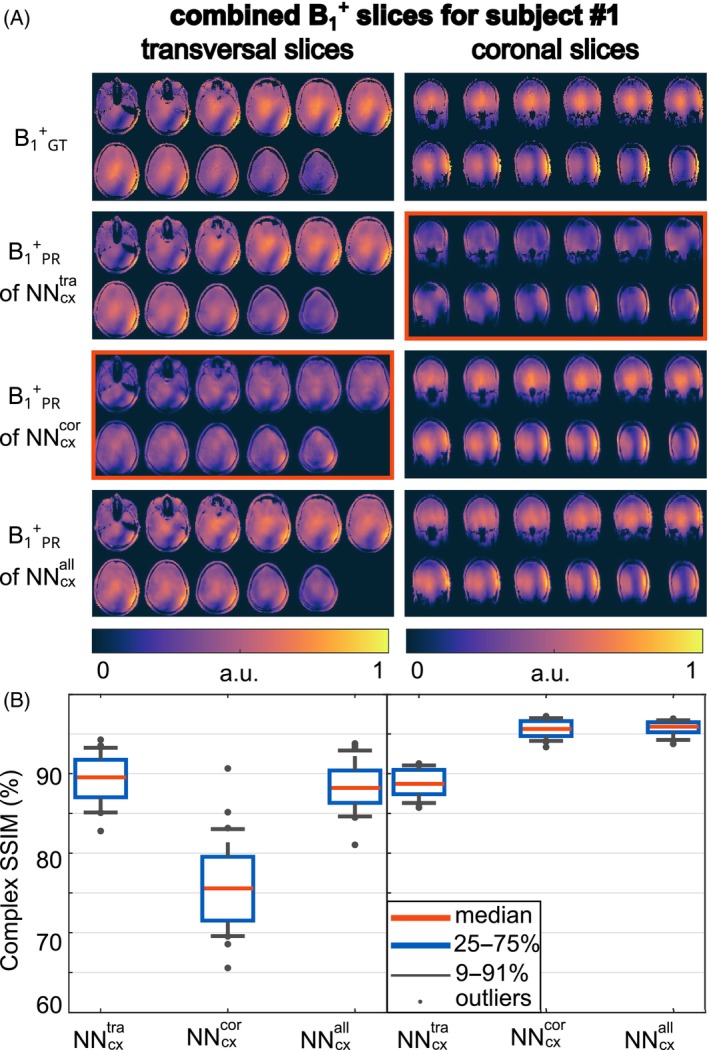
(A) Combined B_1_^+^‐magnitude for the ground truth (GT) B_1_^+^_GT_ compared to the prediction (B_1_^+^_PR_) of different neural networks regarding transversal and coronal slices. ${\mathrm{NN}}_{\mathrm{cx}}^{\mathrm{tra}}$ was trained on axial slices and ${\mathrm{NN}}_{\mathrm{cx}}^{\mathrm{cor}}$ was trained on coronal slices. In contrast, ${\mathrm{NN}}_{\mathrm{cx}}^{\mathrm{all}}$ was trained on axial, sagittal, and coronal slices. Note that the displayed data is not masked. The red boxes indicate a failure to predict the B_1_^+^‐maps. (B) SSIM for the complex‐valued data of the prediction compared to the GT for network configurations ${\mathrm{NN}}_{\mathrm{cx}}^{\mathrm{tra}}$, ${\mathrm{NN}}_{\mathrm{cx}}^{\mathrm{cor}}$, and ${\mathrm{NN}}_{\mathrm{cx}}^{\mathrm{all}}$.

Figure 4A shows the Tx‐channel‐combined B_1_^+^‐magnitude for transversal and coronal slices, comparing the B_1_^+^‐reference to the PR of ${\mathrm{NN}}_{\mathrm{cx}}^{\mathrm{tra}}$, ${\mathrm{NN}}_{\mathrm{cx}}^{\mathrm{cor}}$, and ${\mathrm{NN}}_{\mathrm{cx}}^{\mathrm{all}}$. ${\mathrm{NN}}_{\mathrm{cx}}^{\mathrm{tra}}$ and ${\mathrm{NN}}_{\mathrm{cx}}^{\mathrm{cor}}$ successfully estimated their trained orientations, failing on the other orientations. ${\mathrm{NN}}_{\mathrm{cx}}^{\mathrm{all}}$ accurately predicted all orientations, highlighting the importance of including all orientations in the training process. Figure 4B compares the prediction quality using the SSIM for network configurations ${\mathrm{NN}}_{\mathrm{cx}}^{\mathrm{tra}}$, ${\mathrm{NN}}_{\mathrm{cx}}^{\mathrm{cor}}$, and ${\mathrm{NN}}_{\mathrm{cx}}^{\mathrm{all}}$. For axial slices, ${\mathrm{NN}}_{\mathrm{cx}}^{\mathrm{tra}}$ yields a mean SSIM of 89.3% (range = 82.8%–94.3%), while ${\mathrm{NN}}_{\mathrm{cx}}^{\mathrm{cor}}$ leads to lower mean values of 70.0% (range = 65.6%–90.7%). In contrast, for coronal slices, ${\mathrm{NN}}_{\mathrm{cx}}^{\mathrm{tra}}$ results in lower mean values of 88.7% (range = 85.7%–91.3%), while ${\mathrm{NN}}_{\mathrm{cx}}^{\mathrm{cor}}$ tends to higher values of 95.6% (range = 93.3%–97.2%). ${\mathrm{NN}}_{\mathrm{cx}}^{\mathrm{all}}$ showed comparable accuracy across all orientations with mean SSIM values of 88.5% (range = 81.1%–93.8%) for axial and 95.7% (range = 93.8%–96.9%) for coronal slices. Similar results can be shown for sagittal slices (Figure S13).

### 3.4 In‐vivo application

**FIGURE 5:**
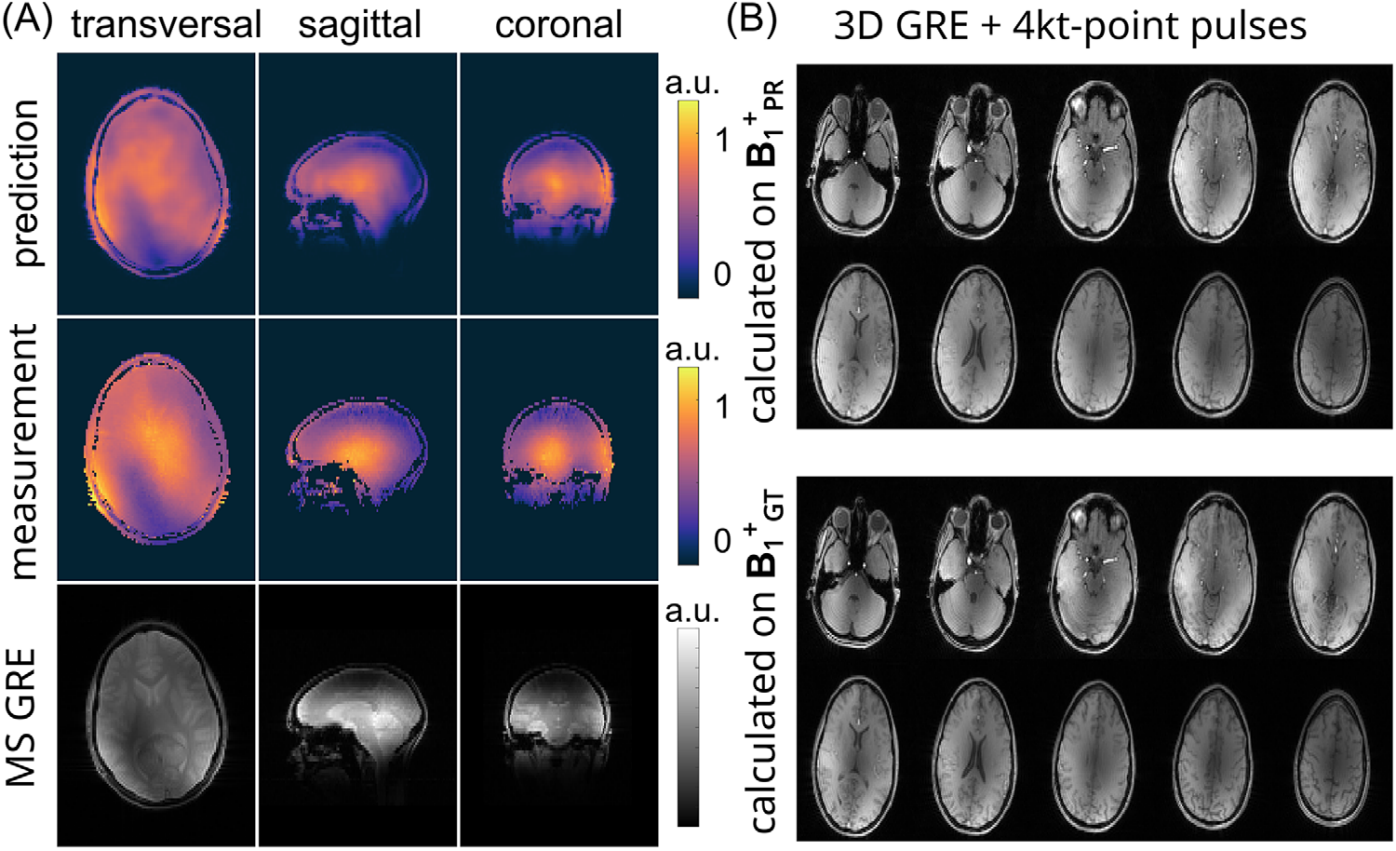
(A) Predicted and measured channel‐combined B_1_^+^‐data compared to the multi‐slice gradient echo (GRE) measurement for all slice orientations for in‐vivo test subject #1. The NN was trained on data of all slice orientations of all 15 subjects. (B) The predicted transversal B_1_^+^‐data was used to calculate four kt‐pulses for a subsequent high‐resolution GRE measurement compared to a high‐resolution GRE acquired with four kt‐pulses calculated on the measured B_1_^+^‐data.

Figure 5A compares the PR and GT channel‐combined B_1_^+^‐data to a multi‐slice GRE measurement for all slice orientations in in‐vivo test subject #1. Qualitatively, a close match between PR, GT channel‐combined B_1_^+^‐data, and GRE images is observed. Note that the receive profiles of the RF coil bias the GRE images. For Figure 5B, the PR transversal B_1_^+^‐data were used to calculate four kt‐point RF pulses for a subsequent high‐resolution GRE measurement. Achieving homogeneous FAs for the whole brain volume demonstrated the feasibility of the suggested DL approach. In comparison, the high‐resolution GRE results are shown when calculating the four kt‐point pulses on the GT. The results for the application in in‐vivo test subject #2 are provided (Figure S14).

## 4 DISCUSSION

This study explored the advantages of employing complex‐valued NNs to estimate B_1_^+^‐maps for eight Tx‐channels based on localizer images acquired in a CP+ ‐mode at 7T. The DL model efficiently predicted multi‐slice B_1_^+^‐maps of the entire brain volume slice‐by‐slice with a computation time of under 1 s per subject. Extending the model to 16 or 32 Tx‐channels increases the computation time approximately linearly with the channel count. Multiple slice orientations were considered during training and ensured a robust PR of B_1_^+^‐maps in different orientations.

Even though B_1_^+^‐data are complex‐valued, most DL‐based approaches in UHF imaging do not support complex representation. For example, Eberhardt et al.^36^ or Kilic et al.^33^ split the complex data into real and imaginary components for two independent real‐valued channels. Plumley et al.^35^ used two separate NNs for magnitude and phase distributions to represent the complex nature of the B_1_^+^‐fields. Other work focused solely on the B_1_^+^‐magnitudes.^29^, ^34^

Our results indicate that complex‐valued NNs can accurately approximate both magnitude and phase data from the localizer images. Localized deviations compared to the GT were only visible for high magnitudes and regions with low SNR or locations with rapidly changing B_1_^+^‐phases. Comparing the complex‐valued ${\mathrm{NN}}_{\mathrm{cx}}^{\mathrm{tra}}$, to real‐valued networks for the same test data revealed comparable prediction quality for the magnitude, indicating a trend toward better prediction quality for the phase (c.f. Figures 1 and S4). However, statistical significance for this trend using the Wilcoxon rank‐sum test investigating the null hypothesis by comparing the prediction quality of the complex‐valued and the corresponding real‐valued networks could not be established, and further investigations are needed.

The feasibility of the DL‐based B_1_^+^‐mapping approach in the context of a subject‐specific pTx was investigated by improving the excitation homogeneity in the human brain using dynamic pTx pulses calculated on the estimated whole‐brain B_1_^+^‐maps. Applying the pulses calculated on the PR to the GT achieved acceptable homogeneous excitation (c.f. Figure 3). However, the CV values were generally higher for pTx pulses calculated on the PR than for pulses calculated on the GT, likely due to residual deviations between PR and GT.

The impact of the slice orientation was analyzed by training NNs on different slice orientations. ${\mathrm{NN}}_{\mathrm{cx}}^{\mathrm{tra}}$, ${\mathrm{NN}}_{\mathrm{cx}}^{\mathrm{sag}}$, and ${\mathrm{NN}}_{\mathrm{cx}}^{\mathrm{cor}}$ estimated B_1_^+^‐maps for their trained orientations with high accuracy but failed for others (c.f. Figures 4 and S13), likely due to varying relative positions of the coil elements. Considering all orientations during training for ${\mathrm{NN}}_{\mathrm{cx}}^{\mathrm{all}}$ enabled accurate prediction of transversal, sagittal, and coronal B_1_^+^‐slices. Further investigations are needed to determine if this applies to any orientations, including double‐oblique orientations.

Limitations include the deliberate use of a custom‐built eight‐channel Tx/Rx transceiver head volume coil, where transmission and receiving are performed using identical coil elements. Thus, B_1_^−^ and B_1_^+^‐profiles were expected to show resembling distributions, possibly enhancing the prediction performance. The impact of a non‐transceiver head coil, for example, the ‘standard’ 8Tx/32Rx 7T head coil from Nova Medical, on the approach's performance is under investigation. Furthermore, the target B_1_^+^‐maps and, thus, the PR were normalized to speed up the learningprocess and to account for changes in the MR system. A conversion to absolute B_1_^+^‐maps is only feasible if identical acquisition conditions are maintained for all training and test datasets; however, additional research is required. Nevertheless, the hybrid B_1_^+^‐mapping technique utilized for data acquisition is unbiased regarding T_1_ relaxation and proton density variations.^37^

Further investigation is necessary to compare the suggested method to rapid acquisition techniques like DREAM. Additionally, the performance of the excitation pulses calculated on DL‐based B_1_^+^‐maps needs to be compared to calibration‐free counterparts like universal pulses. The FA error introduced by the estimated B_1_^+^‐data into subject‐tailored pulses is currently more pronounced than the excitation error of universal pulses in unseen subjects.^13^, ^21^ However, the performance of the presented technique is expected to improve with a larger training library. Compared to the previous work on the human body,^37^ we were able to use less training data, which may be related to the higher SNR, the lack of respiratory motion, similar shapes between subjects, and other factors. Applying the presented approach to the body requires more training data to account for inter‐subject variations and avoid overfitting. Furthermore, patients may need to be included to account for geometrical anomalies.

Combining this work with other DL‐based approaches, for example, for pTx pulse design^30^, ^33^ or physics‐informed DL‐approaches exploiting prior knowledge about the underlying physics,^55^, ^56^ could create a robust RF calibration pipeline for UHF MRI entirely based on DL.

## 5 CONCLUSIONS

In conclusion, this work presents a DL‐based approach to estimate multi‐slice B_1_^+^‐maps from localizers in the human head at 7T. The complex‐valued networks accurately predict B_1_^+^‐distributions in multiple orientations with sufficient quality for dynamic pTx. Although the prediction quality did not improve significantly compared to real‐valued networks, phase‐sensitive applications might benefit from complex‐valued networks due to a trend to an enhanced phase prediction.

## Supporting information


**Figure S1.** Network architecture of the complex‐valued, convolutional neural networks ${\mathrm{NN}}_{\mathrm{cx}}$. Each encoder and decoder stage includes layers with complex convolutions (Conv) with K feature maps and modified rectified linear unit (ModReLU) activations for feature extraction and up−/ down‐sampling. The decoder pipelines are split up per transmission (Tx) channel.
**Figure S2.** (A) Corresponding real‐valued networks (${\mathrm{NN}}_{\text{real}}^{\mathrm{tra},1}$, ${\mathrm{NN}}_{\text{real}}^{\mathrm{tra},2}$, ${\mathrm{NN}}_{\text{real}}^{\mathrm{tra},3}$) to the introduced complex‐valued network (${\mathrm{NN}}_{\mathrm{cx}}^{\mathrm{tra}}$) with increasing trainable parameters by modifying the number of feature maps. The real‐valued networks employ two sequential (3 × 3) convolutions with (1,1) strides for feature extraction, along with an additional (3 × 3) (up‐) convolution using (2,2) strides for up‐ or down‐sampling with rectified linear units (ReLU) as activation functions in each stage. For data processing, the real and imaginary parts of the channel‐wise localizers and B_1_^+^‐maps were separated and concatenated, leading to 128 × 96 × 17 for the input with 8 real, 8 imaginary localizers and one root‐sum‐of‐squares magnitude image and 128 × 96 × 16 for the output with 8 transmission channels. (B) Network architecture (${\mathrm{NN}}_{\text{real}}^{\text{orig}}$) based on the recently introduced real‐valued, convolutional neural network for the thorax applied to the human head. Each encoder and decoder stage includes a max pool layer, two 3 × 3 convolutions with *K* = 32 feature maps, a batch norm (BN), and a leaky ReLU activation function. The architecture is modified (${\mathrm{NN}}_{\text{real}}^{\mathrm{mod}}$) omitting the batch norm and using a ReLU activation function for every convolution layer.
**Figure S3.** Schematical overview of the hybrid approach. Multi‐slice, whole‐brain gradient echo (GRE) localizer images are acquired for different slice orientations with all Tx‐channels transmitting. The acquisition is repeated eight times depending on slice orientation, with only a single Tx‐channel active for each measurement. The Tx‐channel‐wise, orientation‐dependent GRE measurements are merged with a 3D actual flip imaging (AFI) data set obtained for all Tx‐coils active. This results in Tx‐channel‐wise, multi‐slice B_1_^+^‐maps with different slice orientations.
**Figure S4.** Comparison of the prediction quality assessed by the mean deviation for the phase (A), the root mean squared error (RMSE) for the magnitude (B), and complex quantity (C) between the complex‐valued network ${\mathrm{NN}}_{\mathrm{cx}}^{\mathrm{tra}}$, corresponding real‐valued networks (${\mathrm{NN}}_{\text{real}}^{\mathrm{tra},1}$ to ${\mathrm{NN}}_{\text{real}}^{\mathrm{tra},3}$), the previously introduced network for the human thorax (${\mathrm{NN}}_{\text{real}}^{\text{orig}}$) and a modified version for the introduced use case (${\mathrm{NN}}_{\text{real}}^{\mathrm{mod}}$). The networks were tested for 32 transversal slices from three unseen subjects. ${\mathrm{NN}}_{\mathrm{cx}}^{\mathrm{tra}}$ predicted magnitudes with a similar mean relative error of (2.70 ± 2.86)% to ${\mathrm{NN}}_{\text{real}}^{\mathrm{tra},1}$ with (2.68 ± 2.85)% (*p*‐value of 0.90), while indicating a trend towards better prediction quality for the phase with a mean absolute error of (6.70 ± 1.99)° compared to (6.90 ± 2.07)° (*p*‐value of 0.30).
**Figure S5.** Combined B_1_^+^‐maps for subject #1 given for the magnitude and phase (A) and the real and imaginary parts (B) for ${\mathrm{NN}}_{\mathrm{cx}}^{\mathrm{tra}}$. The predicted (B_1_^+^_PR_) and the reference (B_1_^+^_GT_) data are shown for the magnitude and phase of the complex summed‐up data. A mean difference in the phase of (1.90° ± 0.68°), a relative error for the magnitude of (0.94 ± 0.29)%, a relative error for the real part of (1.86 ± 1.84)%, and (1.89 ± 2.23)% for the imaginary part in the brain tissue can be calculated. The multi‐slice data is depicted with a static, phase‐only shim setting, calculated on the center slice of the predicted B_1_^+^‐maps for an optimized efficiency set to a predefined value of 60% to avoid a zero‐phase of the summed‐up B_1_^+^‐data. The best prediction quality (highlighted in blue) was seen for the top slices, whereas the worst performance was found in the bottom slices (marked in green). For subsequent analysis, a slice located in the middle has been selected.
**Figure S6.** Combined B_1_^+^‐maps for unseen Subject #2 and Subject #3 for ${\mathrm{NN}}_{\mathrm{cx}}^{\mathrm{tra}}$. The predicted (B_1_^+^_PR_) and the reference (B_1_^+^_GT_) data are shown for the magnitude and phase of the complex summed‐up data. The multi‐slice data is depicted with a static, phase‐only shim setting, calculated on the center slice of the predicted B_1_^+^‐maps for an optimized efficiency set to a predefined value of 60% to avoid a vanishing phase distribution for the summed‐up data due to the common phase as a reference. for ${\mathrm{NN}}_{\mathrm{cx}}^{\mathrm{tra}}$ yields a PR with a mean relative error for the magnitude of (1.13 ± 0.15) % and (0.59 ± 0.12) % and a mean difference in the phase of (2.92° ± 0.92°) and (1.04° ± 0.35°) for the brain tissue.
**Figure S7.** Combined B_1_^+^‐maps for the unseen subject #1 in a sagittal orientation predicted by ${\mathrm{NN}}_{\mathrm{cx}}^{\mathrm{sag}}$ and a coronal orientation predicted by ${\mathrm{NN}}_{\mathrm{cx}}^{\mathrm{cor}}$. The predicted (B_1_^+^_PR_) and the reference (B_1_^+^_GT_) data are shown for the magnitude and phase of the complex summed‐up data. The multi‐slice data is depicted with a static, phase‐only shim setting, calculated on the center slice of the predicted B_1_^+^‐maps for an optimized efficiency set to a predefined value of 0.60% to avoid a vanishing phase distribution for the summed‐up data due to the common phase as a reference.
**Figure S8.** Channel‐wise B_1_^+^‐magnitude and phase maps for the prediction (PR) of network ${\mathrm{NN}}_{\mathrm{cx}}^{\mathrm{tra}}$ B_1_^+^_PR_ compared to the reference as the ground truth (GT) B_1_^+^_GT_ for the selected inferior slice of unseen subject #1. Only minor deviations are visible in the magnitude and phase for the absolute error ΔB_1_^+^ between PR and GT. Vertical and horizontal profiles through the channel‐wise magnitude maxima for the PR (black) and GT (red) B_1_^+^‐maps are provided for all transmission (Tx) channels. Overall, the prediction qualitatively matches the GT for both the magnitude and the phase.
**Figure S9.** Channel‐wise B_1_^+^‐magnitude and phase maps for the prediction (PR) of network ${\mathrm{NN}}_{\mathrm{cx}}^{\mathrm{tra}}$ B_1_^+^_PR_ compared to the reference as the ground truth (GT) B_1_^+^_GT_ for the exemplary slice of all three test subjects. Only minor deviations are visible in the magnitude and phase for the absolute error ΔB_1_^+^ between PR and GT for all depicted slices. The absolute error ΔB_1_^+^ considered for the magnitude Δ|B_1_^+^| = | B_1_^+^_PR_ ‐ B_1_^+^_GT_ | and phase ∠B_1_^+^ = ∠(B_1_^+^_PR_ / B_1_^+^_GT_), whereas B_1_^+^_PR_ and B_1_^+^_GT_ are complex quantities.
**Figure S10.** Pixel‐wise correlation between the predicted (PR) B_1_^+^‐maps (B_1_^+^_PR_) using ${\mathrm{NN}}_{\mathrm{cx}}^{\mathrm{tra}}$ and the reference as the ground truth (GT) B_1_^+^_GT_ for the magnitude |B_1_^+^| (A) and the phase ∠B_1_^+^ (B) for all Tx‐channels regarding the brain tissue. The phase is unwrapped to avoid phase jumps greater than or equal to π radians. The correlation plots are illustrated for the shown example and the best and worst cases regarding the structure similarity index measure. The axes range between 0 and 1 for the relative magnitude (A) and 0 and 2π for phase (B). The Pearson coefficients ρ are given for every correlation plot. A linear correlation between PR and GT becomes evident, leading to mean Pearson coefficients of ρ = (0.966 ± 0.018) for the magnitude and *ρ* = (0.884 ± 0.142) for the phase. Tx‐channels 1, 2, 6, and 7 exhibit the most significant deviation from linear behavior, resulting in the lowest Pearson coefficients among the cases presented.
**Figure S11.** Combined B_1_^+^‐maps for unseen subject #1 of Fold #3 given for the magnitude and phase for ${\mathrm{NN}}_{\mathrm{cx}}^{\mathrm{tra}}$. The predicted (B_1_^+^_PR_) and the reference (B_1_^+^_GT_) data are shown for the magnitude and phase of the complex summed‐up data. The multi‐slice data is depicted with a static, phase‐only shim setting, calculated on the center slice of the predicted B_1_^+^‐maps for an optimized efficiency set to a predefined value of 60% to avoid a zero‐phase of the summed‐up B_1_^+^‐data. The best prediction quality (highlighted in blue) was seen for the top slices, whereas the worst performance was found in the bottom slices (marked in green). For subsequent analysis, a slice located in the middle has been selected.
**Figure S12.** (A) B_1_^+^‐shimming results for the example slice, best and worst case of unseen subject #1. The shim setting **b**_CP+_ for transmission in a CP+ ‐mode is compared to the homogeneous shim **b**_Hom_ calculated on the prediction B_1_^+^_PR_ of network ${\mathrm{NN}}_{\mathrm{cx}}^{\mathrm{tra}}$ and ground truth B_1_^+^_GT_ and subsequently applied the ground truth. The magnitude of the complex sum (MOS) and phase of the complex sum (POS) over the eight transmission channels are shown. The zero‐phase in the POS in the default case is due to the calculation relative to the phase of the superimposed complex‐valued B_1_^+^‐maps of all Tx‐channels. Optimizing the Tx‐phase by considering the CV as a cost function improves homogeneity in the selected ROI at the expense of a lower magnitude level. (B) Coefficient of variation (CV) in the brain for **b**_CP+_ and **b**_Hom_, independently optimized on the reference data and the predicted B_1_^+^‐maps of the complex (${\mathrm{NN}}_{\mathrm{cx}}^{\mathrm{tra}}$) and real‐valued networks (${\mathrm{NN}}_{\text{real}}^{\mathrm{tra},1}$ to ${\mathrm{NN}}_{\text{real}}^{\mathrm{tra},3}$) and applied to the B_1_^+^_GT_ data of all unseen 32 2D B_1_^+^‐slices of subset #2. Applying **b**_Hom_ to B_1_^+^_GT_ improves the mean CV for all introduced cases calculated on B_1_^+^_PR_, but the CV values are generally higher than applying a **b**_Hom_ optimized on B_1_^+^_GT_.
**Figure S13.** (A) Combined B_1_^+^‐magnitude for the reference (GT) B_1_^+^_GT_ compared to the prediction (B_1_^+^_PR_) of different neural networks regarding sagittal slices. ${\mathrm{NN}}_{\mathrm{cx}}^{\mathrm{tra}}$ was trained on axial slices and ${\mathrm{NN}}_{\mathrm{cx}}^{\mathrm{sag}}$ was trained on sagittal slices. In contrast, ${\mathrm{NN}}_{\mathrm{cx}}^{\mathrm{all}}$ was trained on axial, sagittal, and coronal slices. Note that the displayed data is not masked. The red box indicates a failure to predict the B_1_^+^‐ maps. (B) Structure similarity index measure (SSIM) of the predicted data compared to the GT for network configurations ${\mathrm{NN}}_{\mathrm{cx}}^{\mathrm{tra}}$, ${\mathrm{NN}}_{\mathrm{cx}}^{\mathrm{sag}}$, and ${\mathrm{NN}}_{\mathrm{cx}}^{\mathrm{all}}$.
**Figure S14.** (A) Predicted and measured channel‐combined B_1_^+^‐data compared to the multi‐slice gradient echo (GRE) measurement for all slice orientations in in‐vivo test case #2. The neural network was trained on all slice orientations of all 15 subjects. (B) The predicted transversal B_1_^+^‐data was used to calculate 4 kt‐pulses for a subsequent high‐resolution GRE measurement compared to a high‐resolution GRE acquired with 4 kt‐pulses calculated on the measured B_1_^+^‐data.
**Table S1.** Predicted image quality of ${\mathrm{NN}}_{\mathrm{cx}}^{\mathrm{tra}}$ trained and tested on transversal slices during a 5‐fold cross‐validation. The image quality is assessed using the mean over the fold of the root mean squared error (RMSE) for the magnitude, the mean deviation of the phase, and the structural similarity index measure (SSIM) averaged real and imaginary parts. Fold #2, tested on subset #2, was utilized for further evaluation.
**Table S2.** Predicted image quality of ${\mathrm{NN}}_{\mathrm{cx}}^{\mathrm{sag}}$ trained and tested on sagittal slices during a 5‐fold cross‐validation. The image quality is assessed using the mean over the fold of the root mean squared error (RMSE) for the magnitude, the mean deviation of the phase, and the structural similarity index measure (SSIM) averaged real and imaginary parts. Fold #2, tested on subset #2, was utilized for further evaluation.
**Table S3.** Predicted image quality of ${\mathrm{NN}}_{\mathrm{cx}}^{\mathrm{cor}}$ trained and tested on coronal slices during a 5‐fold cross‐validation. The image quality is assessed using the mean over the fold of the root mean squared error (RMSE) for the magnitude, the mean deviation of the phase, and the structural similarity index measure (SSIM) averaged real and imaginary parts. Fold #2, tested on subset #2, was utilized for further evaluation.
**Table S4.** Predicted image quality of ${\mathrm{NN}}_{\mathrm{cx}}^{\mathrm{all}}$ trained and tested on all slice orientations during a 5‐fold cross‐validation. The image quality is assessed using the mean over the fold of the root mean squared error (RMSE) for the magnitude, the mean deviation of the phase, and the structural similarity index measure (SSIM) averaged real and imaginary parts. Fold #2, tested on subset #2, was utilized for further evaluation.

## Data Availability

The source code, the used neural networks, and an exemplary dataset are provided: https://github.com/felixkrueger90/ComplexB1.
