# Supplementary material for: Deep learning‐based whole‐brain B1 +‐mapping at 7T
Source: Magn Reson Med. 2024 Oct 27;93(4):1700–11. doi: 10.1002/mrm.30359 (PMC11782730; doi:10.1002/mrm.30359)
Supplement: Supplementary file 1 — Figure S1. Network architecture of the complex‐valued, convolutional neural networks NNcx. Each encoder and decoder stage includes layers with complex convolutions (Conv) with K feature maps and modified rectified linear unit (ModReLU) activations for feature extraction and up−/ down‐sampling. The decoder pipelines are split up per transmission (Tx) channel. Figure S2. (A) Corresponding real‐valued networks (NNrealtra,1, NNrealtra,2, NNrealtra,3) to the introduced complex‐valued network (NNcxtra) with increasing trainable parameters by modifying the number of feature maps. The real‐valued networks employ two sequential (3 × 3) convolutions with (1,1) strides for feature extraction, along with an additional (3 × 3) (up‐) convolution using (2,2) strides for up‐ or down‐sampling with rectified linear units (ReLU) as activation functions in each stage. For data processing, the real and imaginary parts of the channel‐wise localizers and B1 +‐maps were separated and concatenated, leading to 128 × 96 × 17 for the input with 8 real, 8 imaginary localizers and one root‐sum‐of‐squares magnitude image and 128 × 96 × 16 for the output with 8 transmission channels. (B) Network architecture (NNrealorig) based on the recently introduced real‐valued, convolutional neural network for the thorax applied to the human head. Each encoder and decoder stage includes a max pool layer, two 3 × 3 convolutions with K = 32 feature maps, a batch norm (BN), and a leaky ReLU activation function. The architecture is modified (NNrealmod) omitting the batch norm and using a ReLU activation function for every convolution layer. Figure S3. Schematical overview of the hybrid approach. Multi‐slice, whole‐brain gradient echo (GRE) localizer images are acquired for different slice orientations with all Tx‐channels transmitting. The acquisition is repeated eight times depending on slice orientation, with only a single Tx‐channel active for each measurement. The Tx‐channel‐wise, orientation‐dependent GRE me [file MRM-93-1700-s001.docx]

# Deep learning-based whole-brain B_1_^+^-mapping at 7T

## Supporting Information

Felix Krueger^1,2^, Christoph Stefan Aigner^1^, Max Lutz^1^, Layla Tabea Riemann^1,3^, Katja Degenhardt^1^, Kimon Hadjikiriakos^1^, Felix Frederik Zimmermann^1^, Kerstin Hammernik^4^, Jeanette Schulz-Menger^5,6,7,8^, Tobias Schaeffter^1,2^, and Sebastian Schmitter^1,9,10^

^1^Physikalisch-Technische Bundesanstalt, Braunschweig and Berlin, Germany

^2^Technische Universität Berlin, Biomedical Engineering, Einstein Centre Digital Future, Berlin, Germany

^3^Institute for Applied Medical Informatics, Center for Experimental Medicine, University Medical Center Hamburg-Eppendorf, Hamburg, Germany

^4^School of Computation, Information and Technology, Technical University of Munich, Munich, Germany

^5^Charité – Universitätsmedizin Berlin, corporate member of Freie Universität Berlin and Humboldt-Universität Zu Berlin, Experimental Clinical Research Center, Berlin, Germany.

^6^Working Group On CMR, Experimental and Clinical Research Center, a joint cooperation between the Charité Medical Faculty and the Max-Delbrueck Center for Molecular Medicine, Berlin, Germany

^7^DZHK (German Centre for Cardiovascular Research), partner site Berlin, Berlin, Germany.

^8^Department of Cardiology and Nephrology, HELIOS Hospital Berlin-Buch, Berlin, Germany.

^9^University of Minnesota, Center for Magnetic Resonance Research, Minneapolis, MN, United States

^10^Medical Physics in Radiology, German Cancer Research Center (DKFZ), Heidelberg, Germany

Correspondence address: Felix Krüger, Physikalisch-Technische Bundesanstalt, Abbestr. 2 – 12, 10587 Berlin, Germany. E-mail: felix.krueger@ptb.de.

## Supporting Figures


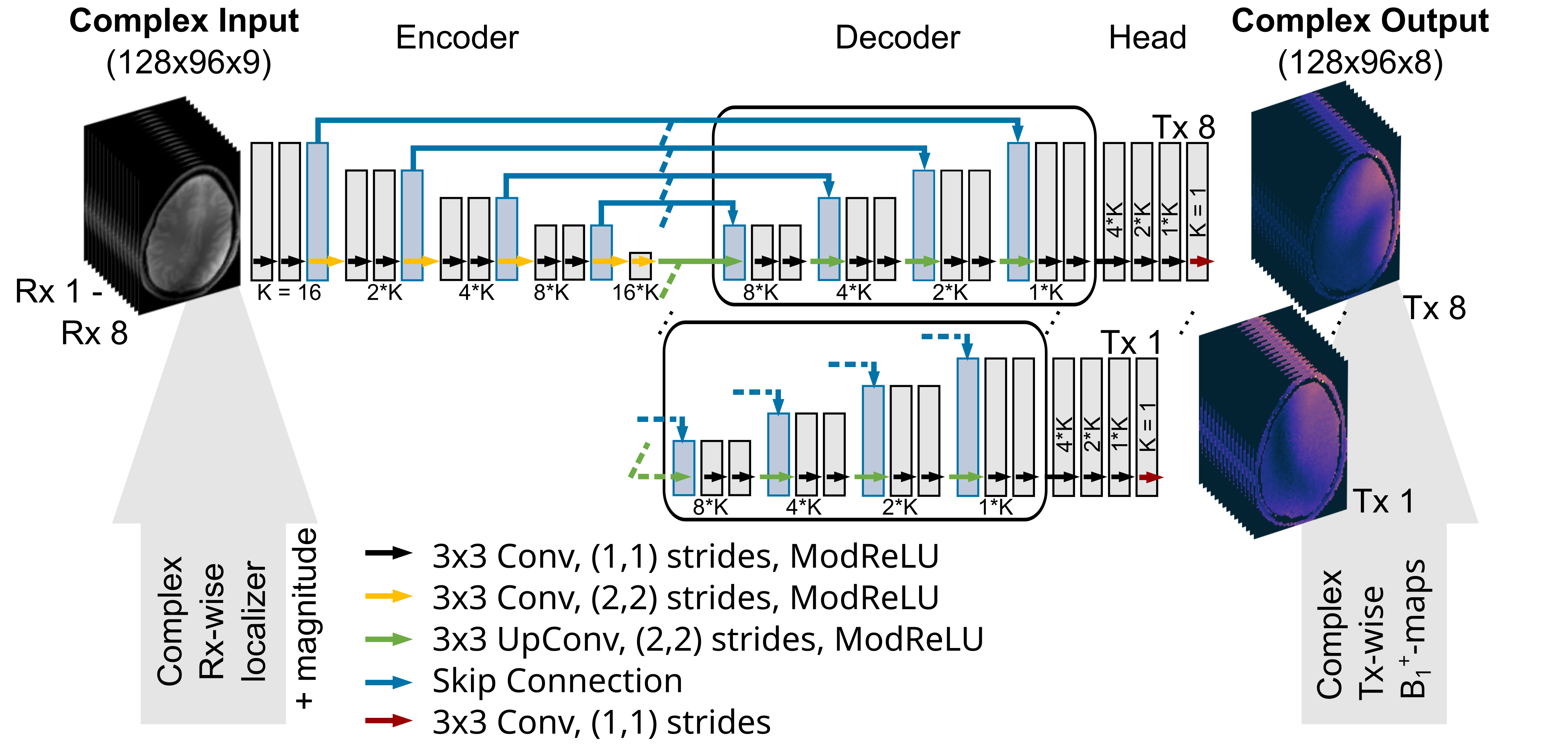


Figure S1: Network architecture of the complex-valued, convolutional neural networks $\mathrm{NN}_{\mathrm{cx}}$. Each encoder and decoder stage includes layers with complex convolutions (Conv) with K feature maps and modified rectified linear unit (ModReLU) activations for feature extraction and up-/ down-sampling. The decoder pipelines are split up per transmission (Tx) channel.


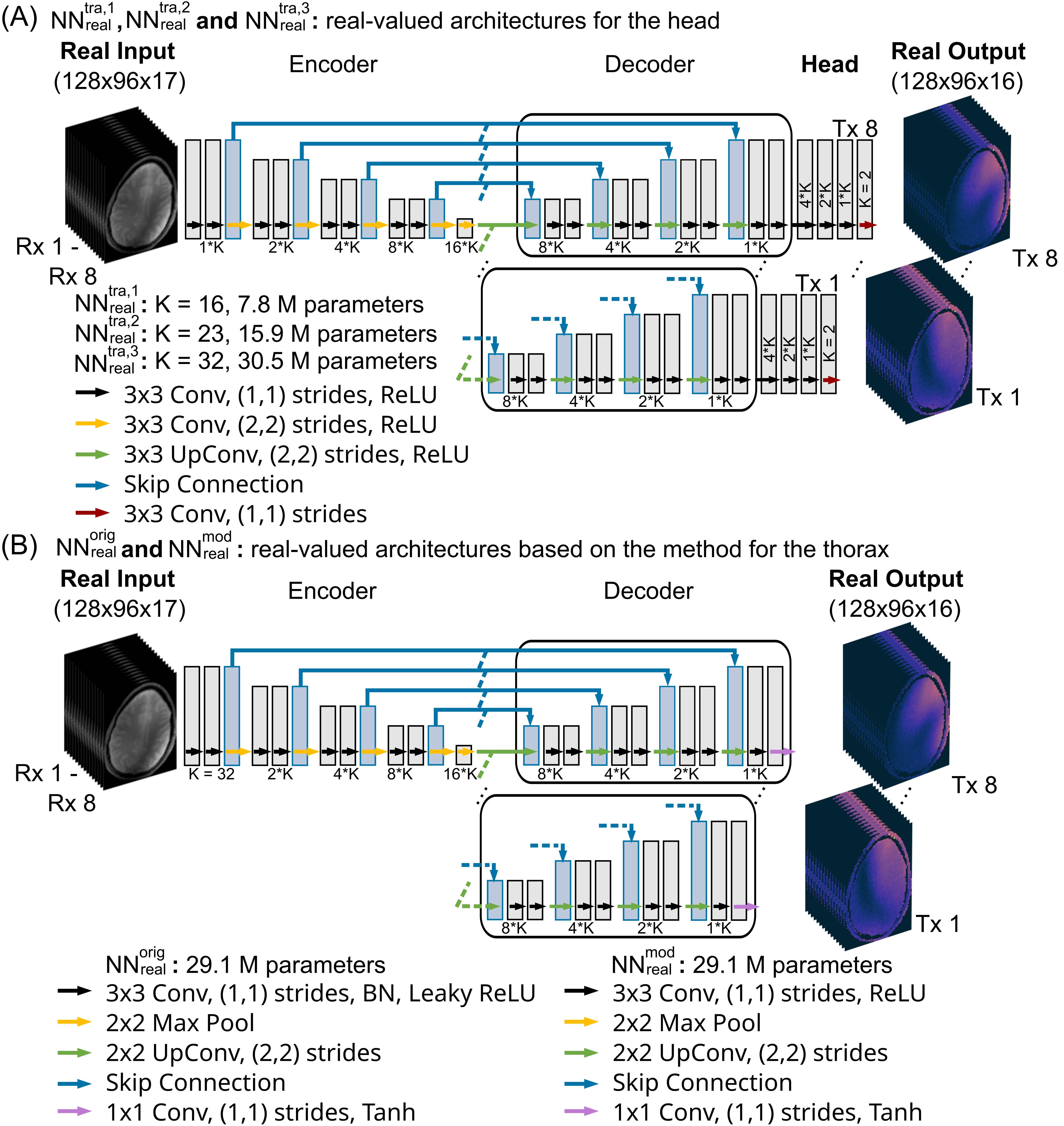


Figure S2: (A) Corresponding real-valued networks ($\mathrm{NN}_{\mathrm{real}}^{tra,1}$, $\mathrm{NN}_{\mathrm{real}}^{tra,2}$, $\mathrm{NN}_{\mathrm{real}}^{tra,3}$) to the introduced complex-valued network ($\mathrm{NN}_{\mathrm{cx}}^{\mathrm{tra}}$) with increasing trainable parameters by modifying the number of feature maps. The real-valued networks employ two sequential (3x3) convolutions with (1,1) strides for feature extraction, along with an additional (3x3) (up-) convolution using (2,2) strides for up- or down-sampling with rectified linear units (ReLU) as activation functions in each stage. For data processing, the real and imaginary parts of the channel-wise localizers and B_1_^+^-maps were separated and concatenated, leading to 128x96x17 for the input with 8 real, 8 imaginary localizers and one root-sum-of-squares magnitude image and 128x96x16 for the output with 8 transmission channels. (B) Network architecture ($\mathrm{NN}_{\mathrm{real}}^{\mathrm{orig}}$) based on the recently introduced real-valued, convolutional neural network for the thorax applied to the human head. Each encoder and decoder stage includes a max pool layer, two 3×3 convolutions with K=32 feature maps, a batch norm (BN), and a leaky ReLU activation function. The architecture is modified ($\mathrm{NN}_{\mathrm{real}}^{\mathrm{mod}}$) omitting the batch norm and using a ReLU activation function for every convolution layer.


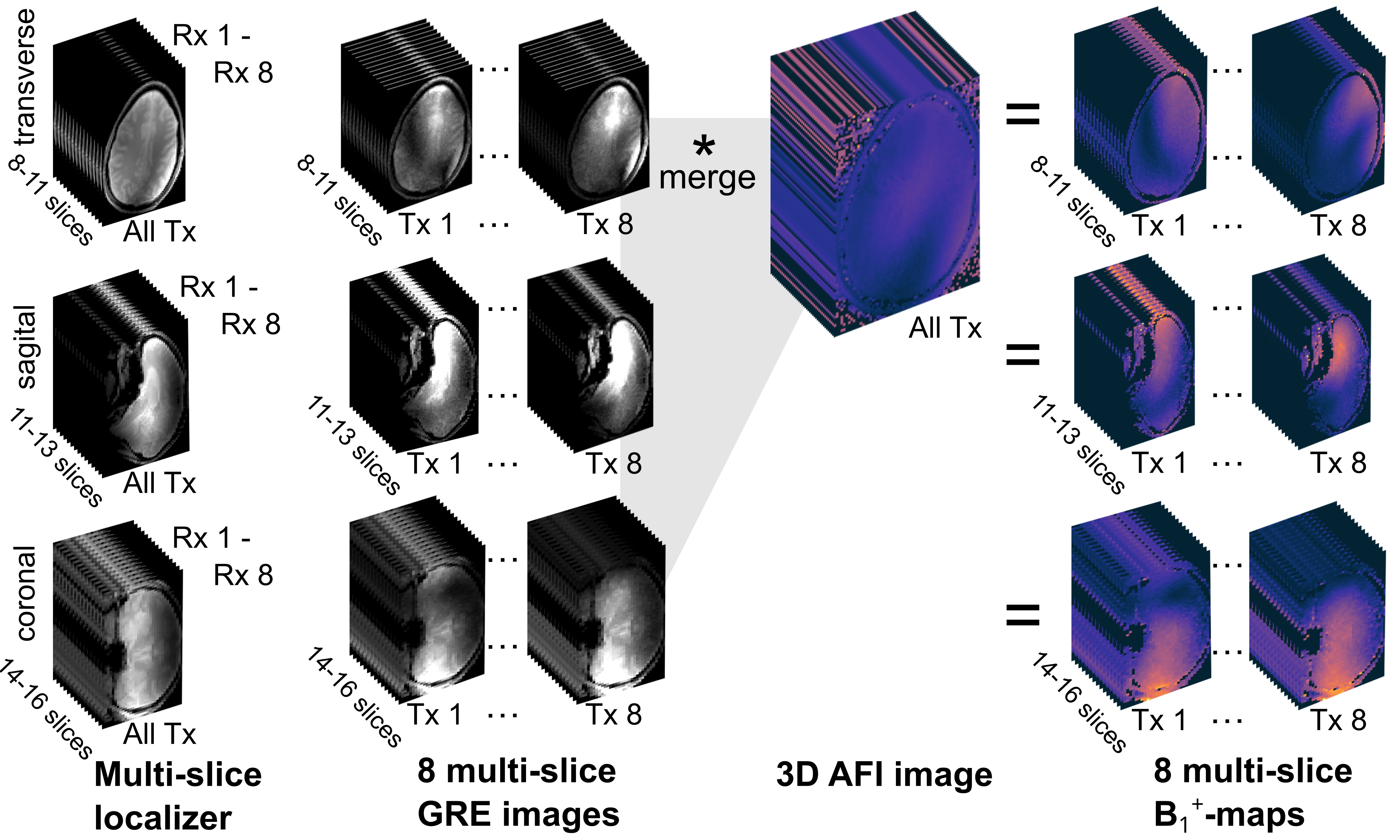


Figure S3: Schematical overview of the hybrid approach. Multi-slice, whole-brain gradient echo (GRE) localizer images are acquired for different slice orientations with all Tx-channels transmitting. The acquisition is repeated eight times depending on slice orientation, with only a single Tx-channel active for each measurement. The Tx-channel-wise, orientation-dependent GRE measurements are merged with a 3D actual flip imaging (AFI) data set obtained for all Tx-coils active. This results in Tx-channel-wise, multi-slice B_1_^+^-maps with different slice orientations.


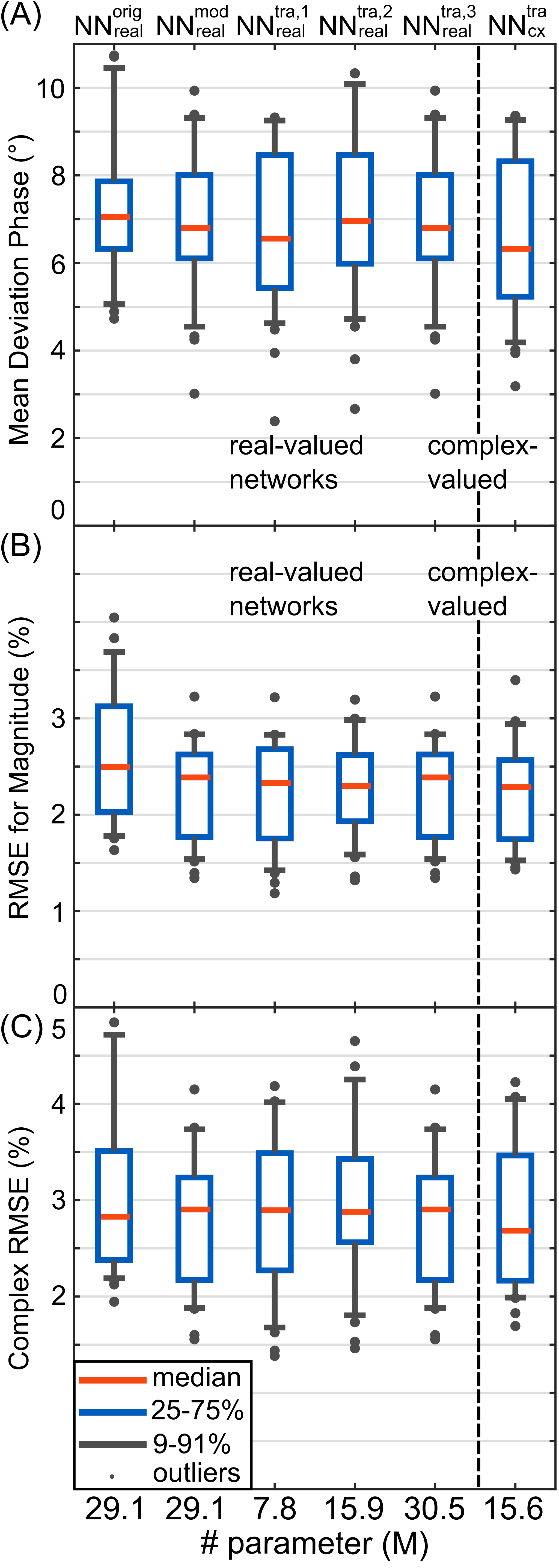


Figure S4: Comparison of the prediction quality assessed by the mean deviation for the phase (A), the root mean squared error (RMSE) for the magnitude (B), and complex quantity (C) between the complex-valued network $\mathrm{NN}_{\mathrm{cx}}^{\mathrm{tra}}$, corresponding real-valued networks ($\mathrm{NN}_{\mathrm{real}}^{tra,1}$ to $\mathrm{NN}_{\mathrm{real}}^{tra,3}$), the previously introduced network for the human thorax ($\mathrm{NN}_{\mathrm{real}}^{\mathrm{orig}}$) and a modified version for the introduced use case ($\mathrm{NN}_{\mathrm{real}}^{\mathrm{mod}}$). The networks were tested for 32 transversal slices from three unseen subjects. $\mathrm{NN}_{\mathrm{cx}}^{\mathrm{tra}}$ predicted magnitudes with a similar mean relative error of (2.70±2.86)% to $\mathrm{NN}_{\mathrm{real}}^{tra,1}$ with (2.68±2.85)% (p-value of 0.90), while indicating a trend towards better prediction quality for the phase with a mean absolute error of (6.70±1.99)° compared to (6.90±2.07)° (p-value of 0.30).


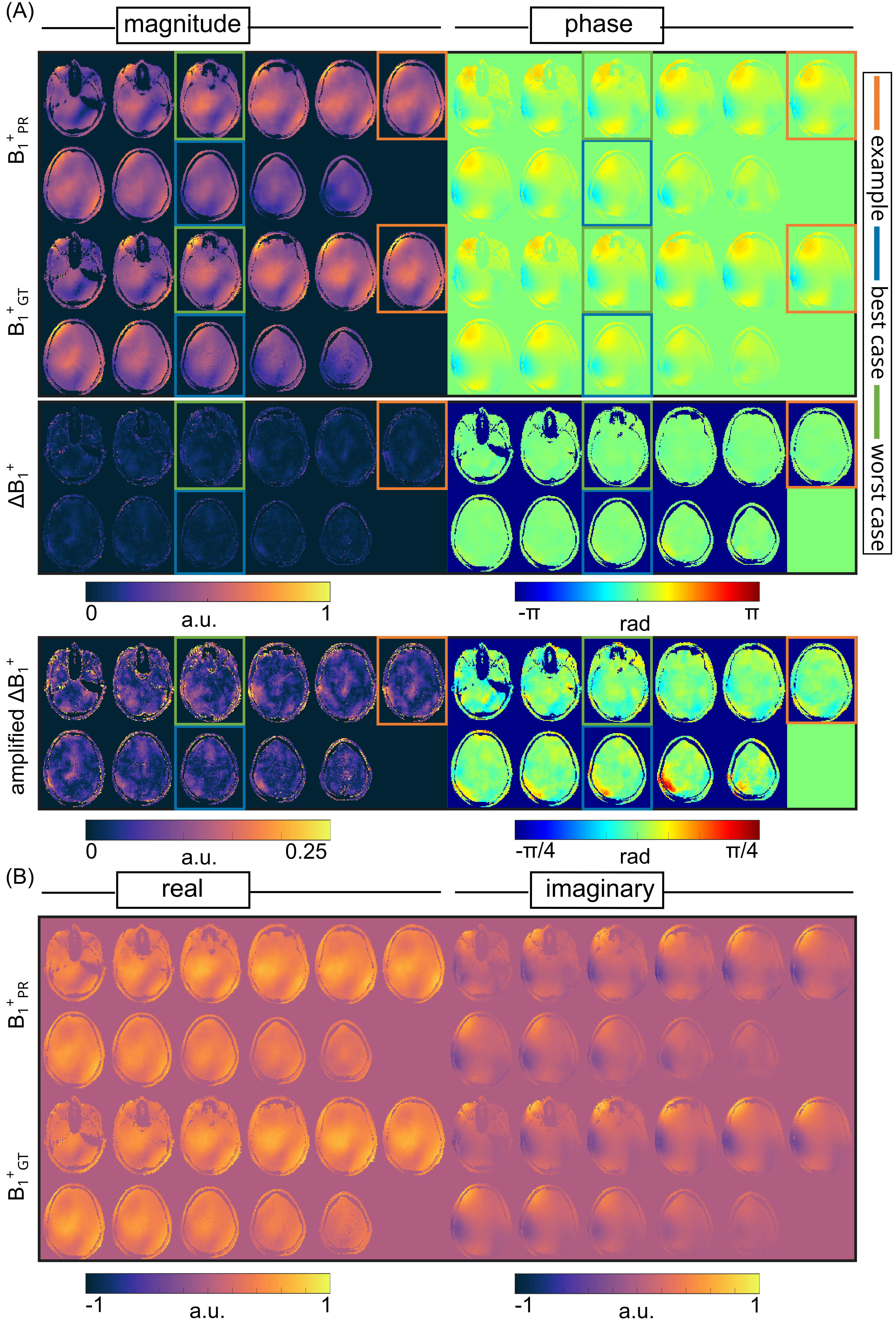


Figure S5: Combined B_1_^+^-maps for unseen subject #1 given for the magnitude and phase (A) and the real and imaginary parts (B) for $\mathrm{NN}_{\mathrm{cx}}^{\mathrm{tra}}$. The predicted (B_1_^+^_PR_), the reference (B_1_^+^_GT_) data, and the absolute differences ΔB_1_^+^ are shown for the magnitude and phase of the complex summed-up data. A mean difference in the phase of (1.90°±0.68°), a relative error for the magnitude of (0.94±0.29)%, a relative error for the real part of (1.86±1.84)%, and (1.89±2.23)% for the imaginary part in the brain tissue can be calculated. The multi-slice data is depicted with a static, phase-only shim setting, calculated on the center slice of the predicted B_1_^+^-maps for an optimized efficiency set to a predefined value of 60% to avoid a zero-phase of the summed-up B_1_^+^-data. The best prediction quality (highlighted in blue) was seen for the top slices, whereas the worst performance was found in the bottom slices (marked in green). A slice in the middle (orange) was selected for subsequent analysis.


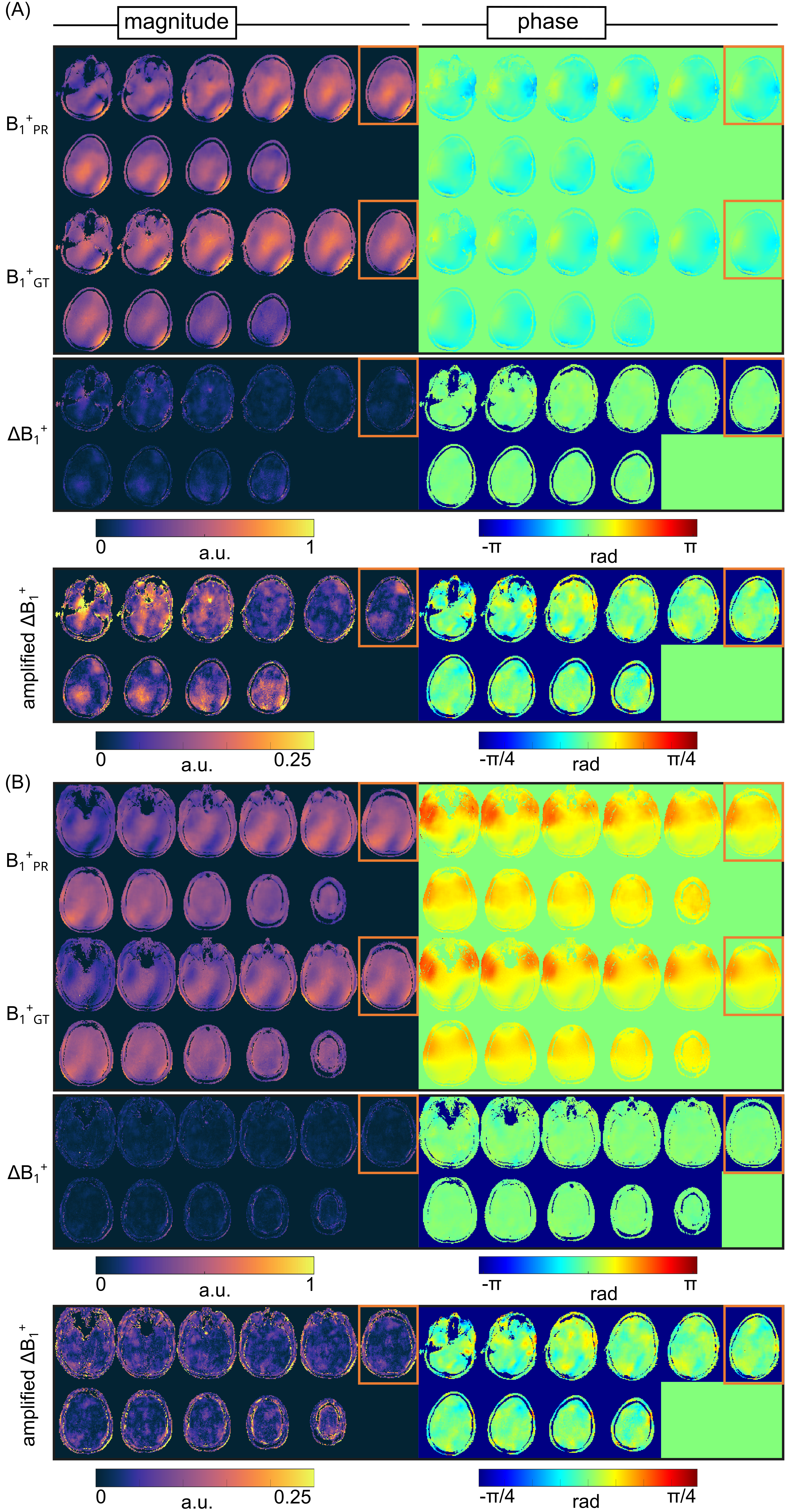


Figure S6 Combined B_1_^+^-maps for unseen subject #2 and subject #3 for for $\mathrm{NN}_{\mathrm{cx}}^{\mathrm{tra}}$. The predicted (B_1_^+^_PR_), the reference (B_1_^+^_GT_) data, and the absolute differences ΔB_1_^+^ are shown for the magnitude and phase of the complex summed-up data. The multi-slice data is depicted with a static, phase-only shim setting, calculated on the center slice of the predicted B_1_^+^-maps for an optimized efficiency set to a predefined value of 60% to avoid a vanishing phase distribution for the summed-up data due to the common phase as a reference. A slice located in the middle (orange) was selected as an example. $\mathrm{NN}_{\mathrm{cx}}^{\mathrm{tra}}$ yields a PR with a mean relative error for the magnitude of (1.13±0.15) % and (0.59±0.12) % and a mean difference in the phase of (2.92°±0.92°) and (1.04°±0.35°) for the brain tissue.


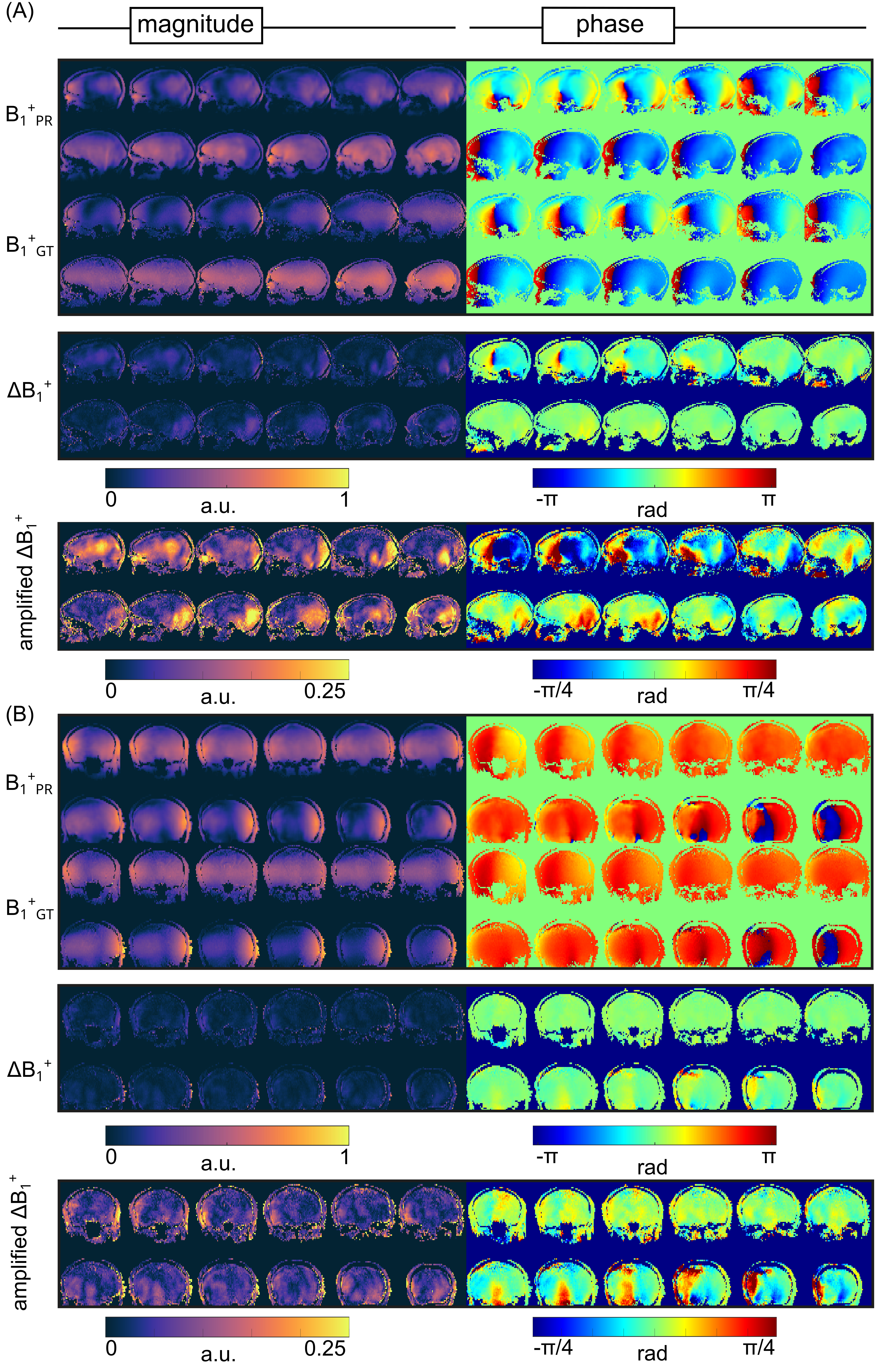


Figure S7: Combined B_1_^+^-maps for unseen subject #1 in a sagittal orientation predicted by $\mathrm{NN}_{\mathrm{cx}}^{\mathrm{sag}}$ and a coronal orientation predicted by $\mathrm{NN}_{\mathrm{cx}}^{\mathrm{cor}}$. The predicted (B_1_^+^_PR_), the reference (B_1_^+^_GT_) data, and the absolute differences ΔB_1_^+^ are shown for the magnitude and phase of the complex summed-up data. The multi-slice data is depicted with a static, phase-only shim setting, calculated on the center slice of the predicted B_1_^+^-maps for an optimized efficiency set to a predefined value of 60% to avoid a vanishing phase distribution for the summed-up data due to the common phase as a reference.


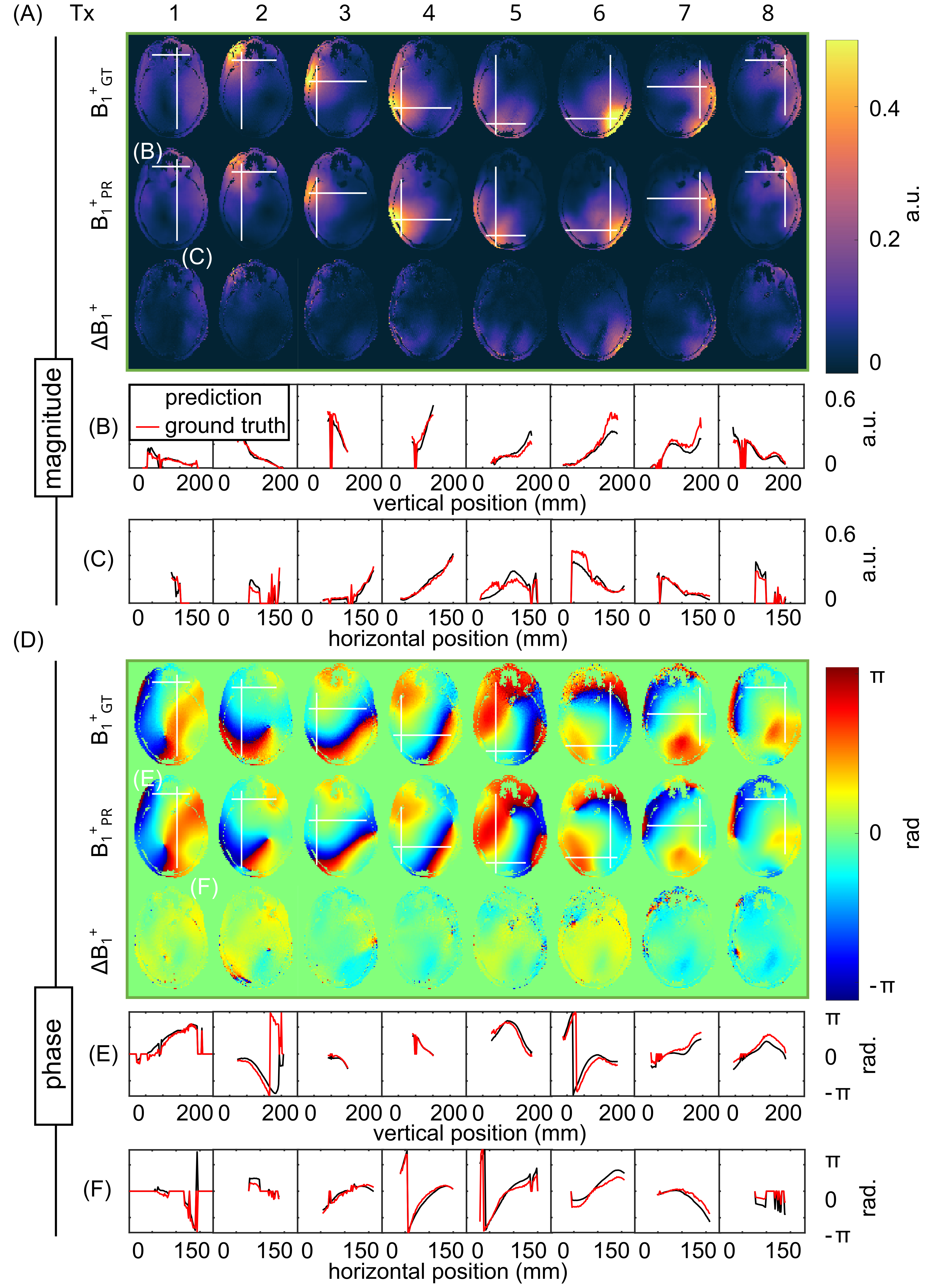


Figure S8: Channel-wise B_1_^+^-magnitude and phase maps for the prediction (PR) of network $\mathrm{NN}_{\mathrm{cx}}^{\mathrm{tra}}$ B_1_^+^_PR_ compared to the reference as the ground truth (GT) B_1_^+^_GT_ for the selected inferior slice of unseen subject #1. Only minor deviations are visible in the magnitude and phase for the absolute error ΔB_1_^+^ for magnitude and phase between PR and GT. Vertical and horizontal profiles through the channel-wise magnitude maxima for the PR (black) and GT (red) B_1_^+^-maps are provided for all transmission (Tx) channels. Overall, the prediction qualitatively matches the GT for both the magnitude and the phase.


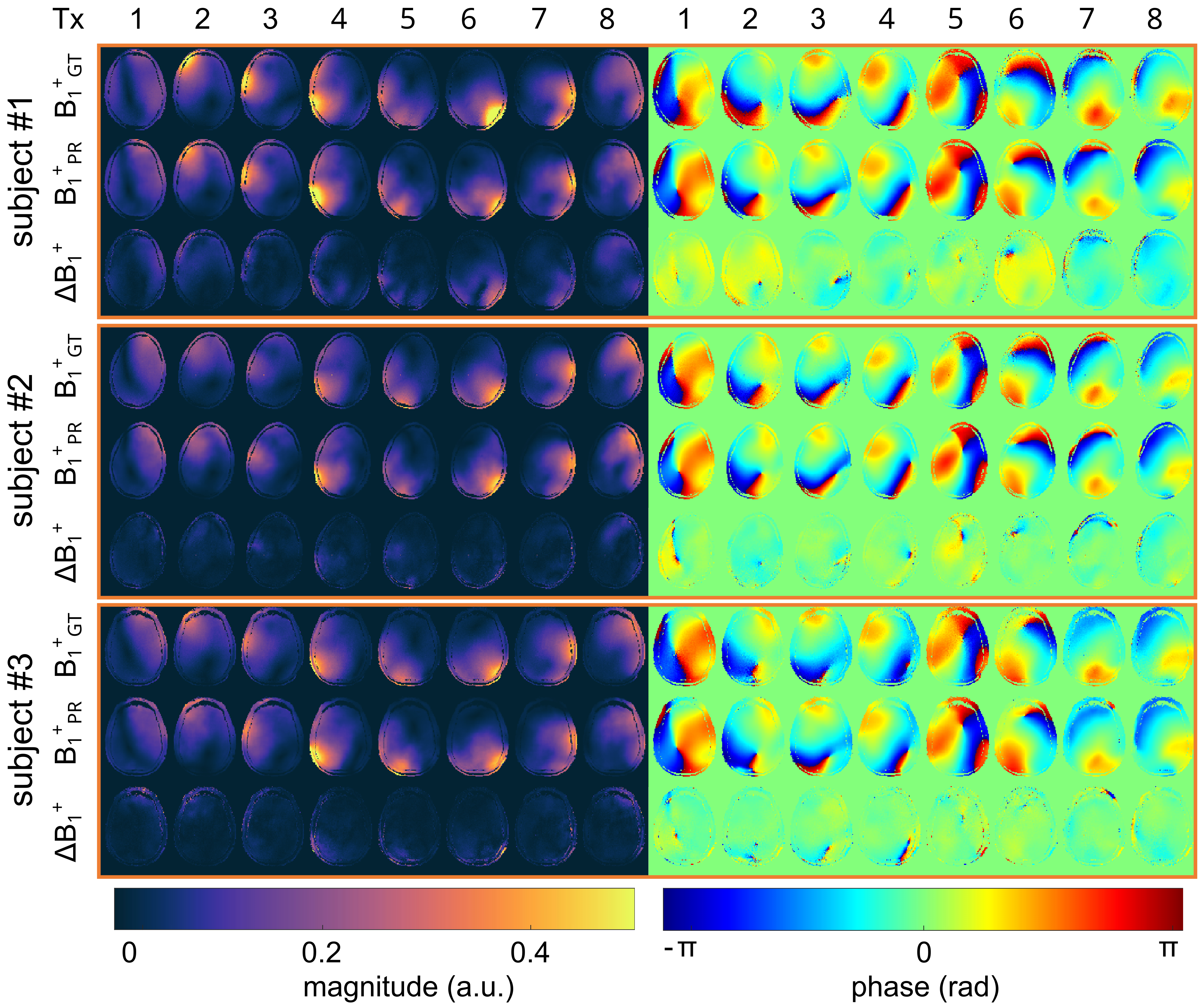


Figure S9: Channel-wise B_1_^+^-magnitude and phase maps for the prediction (PR) of network $\mathrm{NN}_{\mathrm{cx}}^{\mathrm{tra}}$ B_1_^+^_PR_ compared to the reference as the ground truth (GT) B_1_^+^_GT_ for the exemplary slice of all three test subjects. Only minor deviations are visible in the magnitude and phase for the absolute error ΔB_1_^+^ between PR and GT for all depicted slices. The absolute error ΔB_1_^+^ considered for the magnitude Δ|B_1_^+^| = | B_1_^+^_PR_ - B_1_^+^_GT_ | and phase ∠B_1_^+^ = ∠( B_1_^+^_PR_ / B_1_^+^_GT_ ), whereas B_1_^+^_PR_ and B_1_^+^_GT_ are complex quantities.


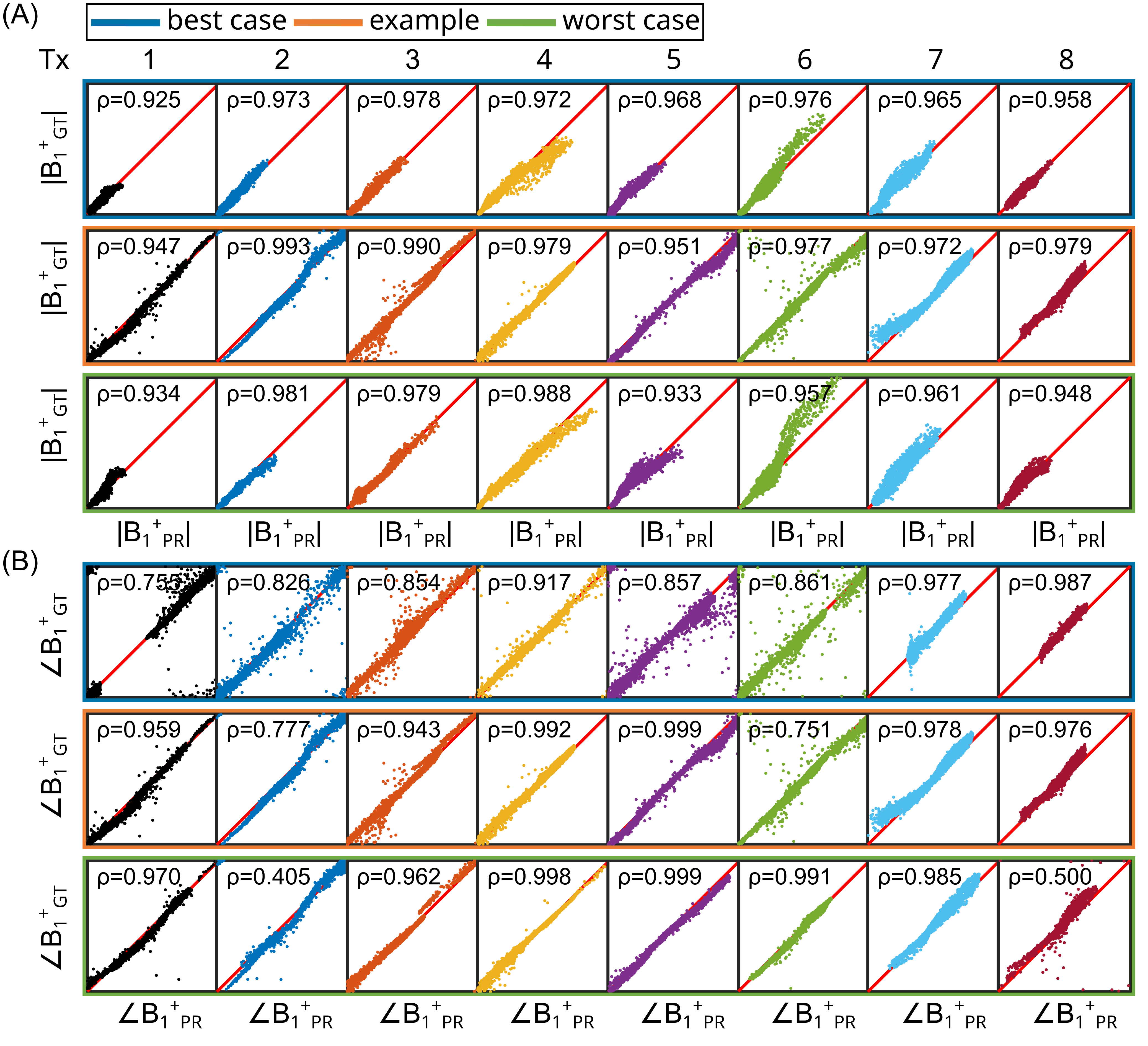


Figure S10: Pixel-wise correlation between the predicted (PR) B_1_^+^-maps (B_1_^+^_PR_) using $\mathrm{NN}_{\mathrm{cx}}^{\mathrm{tra}}$ and the reference as the ground truth (GT) B_1_^+^_GT_ for the magnitude |B_1_^+^| (A) and the phase ∠B_1_^+^ (B) for all Tx-channels regarding the brain tissue. The phase is unwrapped to avoid phase jumps greater than or equal to π radians. The correlation plots are illustrated for the shown example and the best and worst cases regarding the structure similarity index measure. The axes range between 0 and 1 for the relative magnitude (A) and 0 and 2π for phase (B). The Pearson coefficients ρ are given for every correlation plot. A linear correlation between PR and GT becomes evident, leading to mean Pearson coefficients of ρ = (0.966±0.018) for the magnitude and ρ = (0.884±0.142) for the phase. Tx-channels 1, 2, 6, and 7 exhibit the most significant deviation from linear behavior, resulting in the lowest Pearson coefficients among the cases presented.


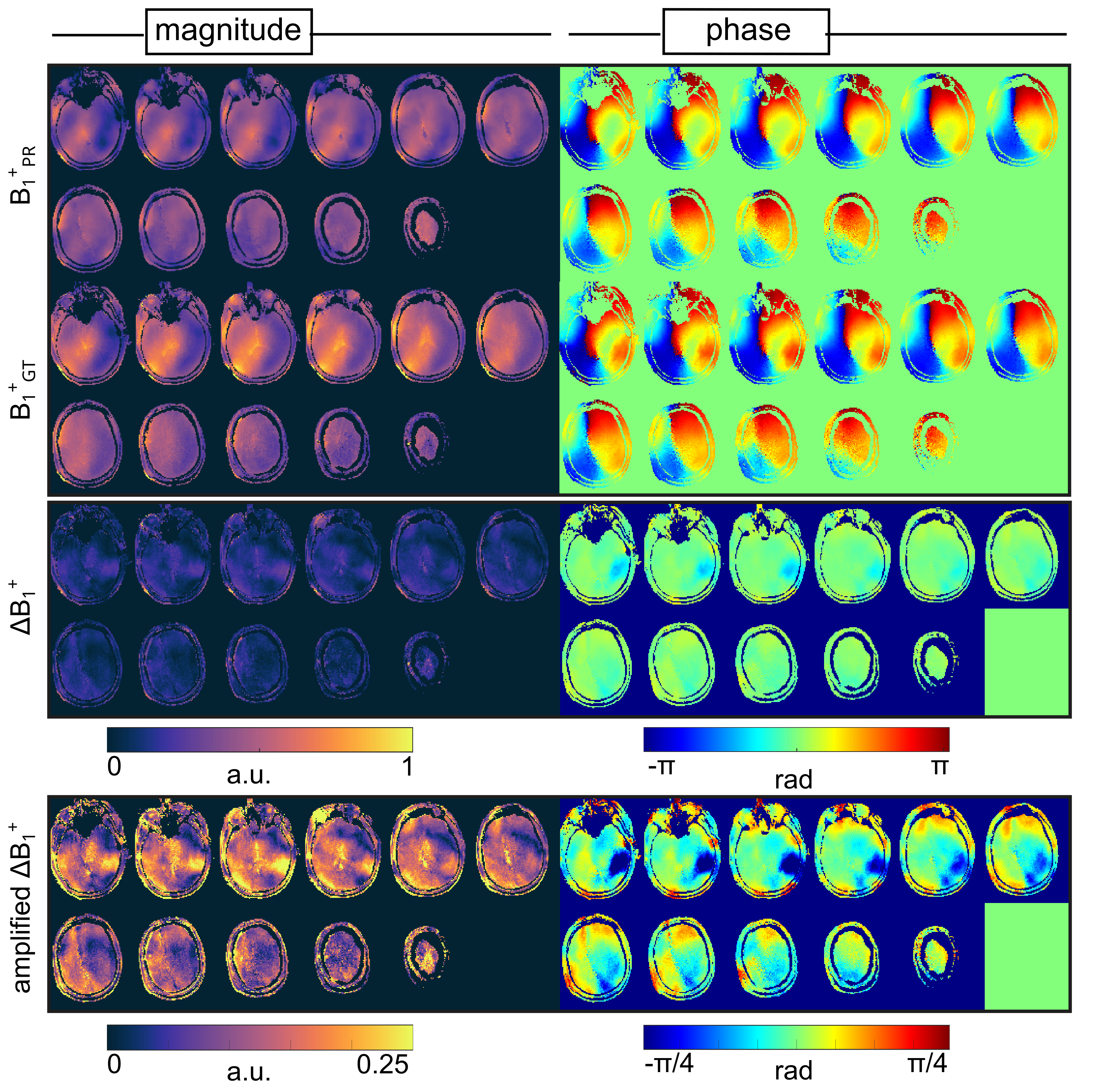


Figure S11: Combined B_1_^+^-maps for unseen subject #1 of Fold #3 given for the magnitude and phase for $\mathrm{NN}_{\mathrm{cx}}^{\mathrm{tra}}$. The predicted (B_1_^+^_PR_), the reference (B_1_^+^_GT_) data, and the absolute error ΔB_1_^+^ are shown for the magnitude and phase of the complex summed-up data. The multi-slice data is depicted with a static, phase-only shim setting, calculated on the center slice of the predicted B_1_^+^-maps for an optimized efficiency set to a predefined value of 60% to avoid a zero-phase of the summed-up B_1_^+^-data. The best prediction quality (highlighted in blue) was seen for the top slices, whereas the worst performance was found in the bottom slices (marked in green). For subsequent analysis, a slice located in the middle has been selected.


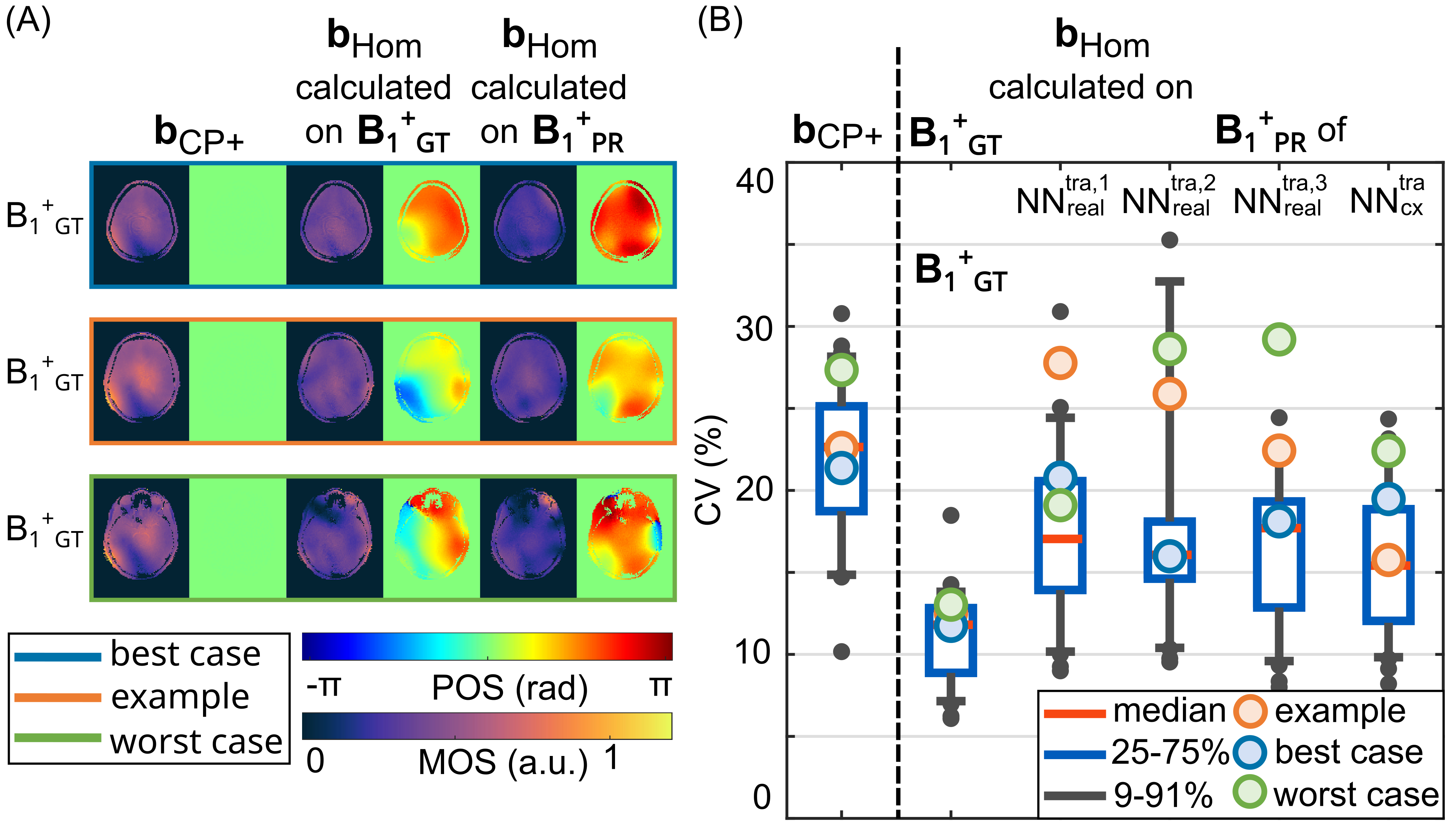


Figure S12: (A) B_1_^+^-shimming results for the example slice, best and worst case of unseen subject #1. The shim setting **b**_CP+_ for transmission in a CP+-mode is compared to the homogeneous shim **b**_Hom_ calculated on the prediction B_1_^+^_PR_ of network $\mathrm{NN}_{\mathrm{cx}}^{\mathrm{tra}}$ and ground truth B_1_^+^_GT_ and subsequently applied the ground truth. The magnitude of the complex sum (MOS) and phase of the complex sum (POS) over the eight transmission channels are shown. The zero-phase in the POS in the default case is due to the calculation relative to the phase of the superimposed complex-valued B_1_^+^-maps of all Tx-channels. Optimizing the Tx-phase by considering the CV as a cost function improves homogeneity in the selected ROI at the expense of a lower magnitude level. (B) Coefficient of variation (CV) in the brain for **b**_CP+_ and **b**_Hom_, independently optimized on the reference data and the predicted B_1_^+^-maps of the complex ($\mathrm{NN}_{\mathrm{cx}}^{\mathrm{tra}}$) and real-valued networks ($\mathrm{NN}_{\mathrm{real}}^{tra,1}$ to $\mathrm{NN}_{\mathrm{real}}^{tra,3}$) and applied to the B_1_^+^_GT_ data of all unseen 32 2D B_1_^+^-slices of subset #2. Applying **b**_Hom_ to B_1_^+^_GT_ improves the mean CV for all introduced cases calculated on B_1_^+^_PR,_ but the CV values are generally higher than applying a **b**_Hom_ optimized on B_1_^+^_GT_.


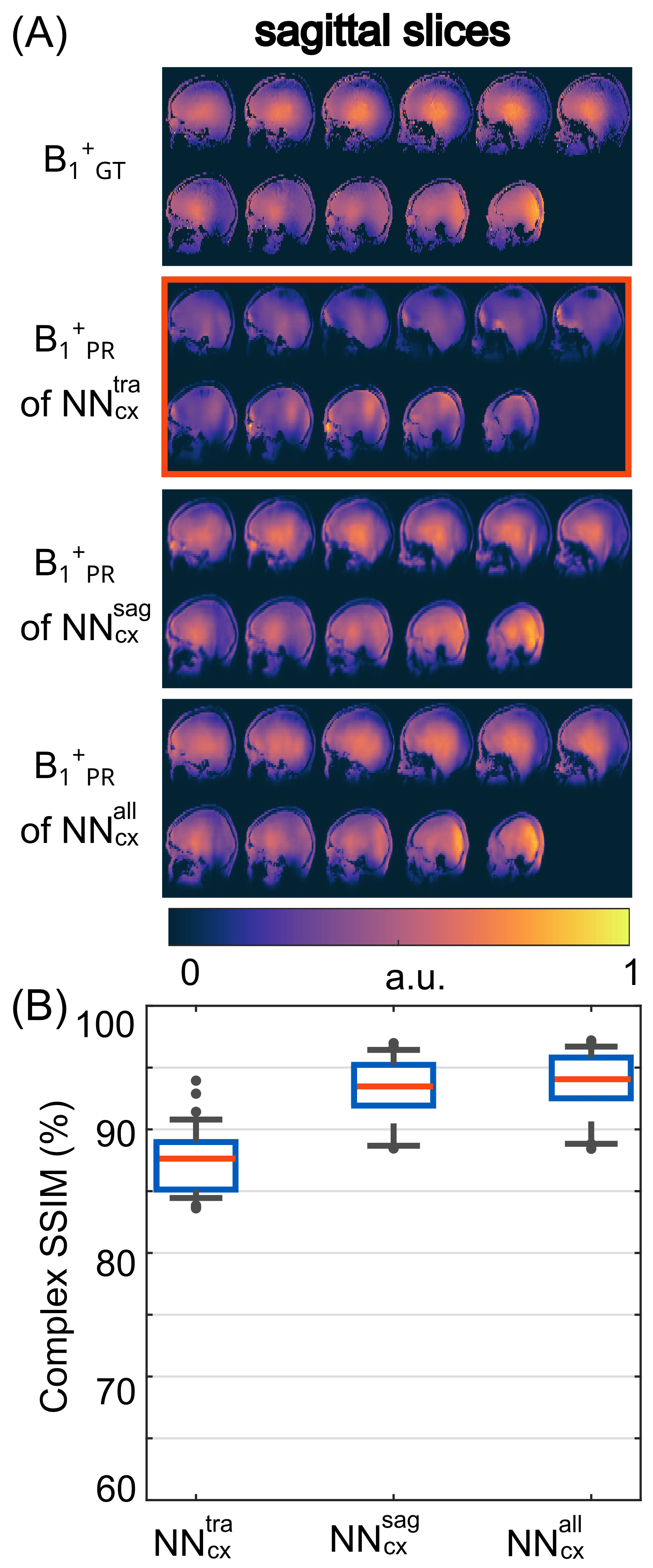
Figure S13: (A) Combined B_1_^+^-magnitude for the reference (GT) B_1_^+^_GT_ compared to the prediction (B_1_^+^_PR_) of different neural networks regarding sagittal slices. $\mathrm{NN}_{\mathrm{cx}}^{\mathrm{tra}}$ was trained on axial slices and $\mathrm{NN}_{\mathrm{cx}}^{\mathrm{sag}}$ was trained on sagittal slices. In contrast, $\mathrm{NN}_{\mathrm{cx}}^{\mathrm{all}}$ was trained on axial, sagittal, and coronal slices. Note that the displayed data is not masked. The red box indicates a failure to predict the B_1_^+^- maps. (B) Structure similarity index measure (SSIM) of the predicted data compared to the GT for network configurations $\mathrm{NN}_{\mathrm{cx}}^{\mathrm{tra}}$, $\mathrm{NN}_{\mathrm{cx}}^{\mathrm{sag}}$, and $\mathrm{NN}_{\mathrm{cx}}^{\mathrm{all}}$.


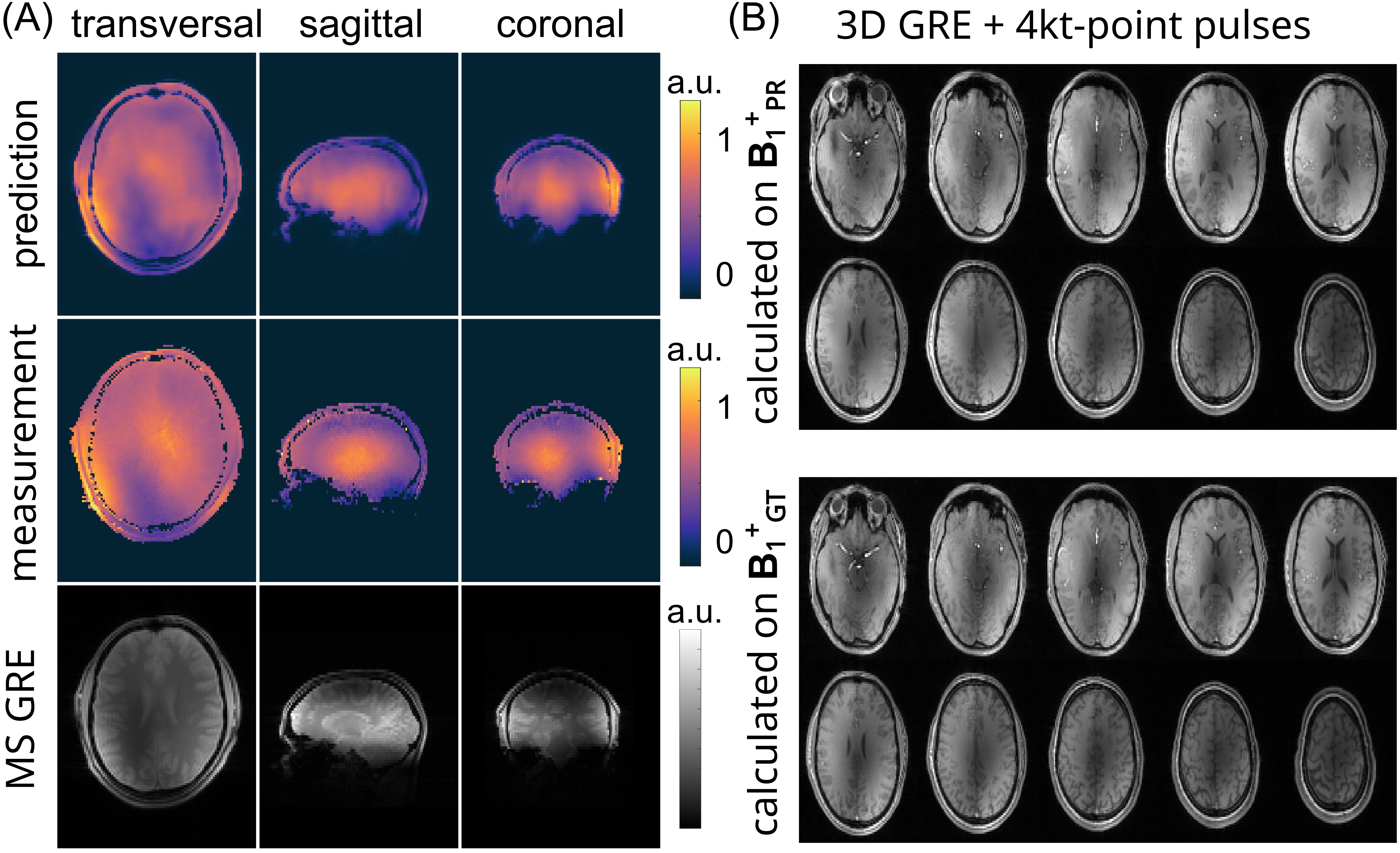
 Figure S14: (A) Predicted and measured channel-combined B_1_^+^-data compared to the multi-slice gradient echo (GRE) measurement for all slice orientations in in-vivo test case #2. The neural network was trained on all slice orientations of all 15 subjects. (B) The predicted transversal B_1_^+^-data was used to calculate 4 kt-pulses for a subsequent high-resolution GRE measurement compared to a high-resolution GRE acquired with 4 kt-pulses calculated on the measured B_1_^+^-data.

## Supporting Tables

Table S1: Predicted image quality of $\mathrm{NN}_{\mathrm{cx}}^{\mathrm{tra}}$ trained and tested on transversal slices during a 5-fold cross-validation. The image quality is assessed using the mean over the fold of the root mean squared error (RMSE) for the magnitude, the mean deviation of the phase, and the structural similarity index measure (SSIM) averaged real and imaginary parts. Fold #2, tested on subset #2, was utilized for further evaluation.

| **Fold of** $\mathrm{NN}_{\mathrm{cx}}^{\mathrm{tra}}$ | **Tested on** | **RMSE of Magnitude**  mean ± std (%) | **Mean Deviation Phase**  mean ± std (°) | **SSIM for Complex**  mean ± std (%) |
| --- | --- | --- | --- | --- |
| #1 | Subset #1 | 2.597 ± 0.926 | 6.581 ± 1.533 | 89.781 ± 2.587 |
| **#2** | **Subset #2** | **2.395 ± 2.894** | **4.485 ± 1.435** | **92.936 ± 2.570** |
| #3 | Subset #3 | 3.077 ± 1.235 | 9.073 ± 3.948 | 85.690 ± 6.423 |
| #4 | Subset #4 | 2.484 ± 0.415 | 6.475 ± 1.504 | 89.595 ± 1.471 |
| #5 | Subset #5 | 2.231 ± 0.405 | 6.925 ± 2.005 | 88.884 ± 2.884 |

Table S2: Predicted image quality of $\mathrm{NN}_{\mathrm{cx}}^{\mathrm{sag}}$ trained and tested on sagittal slices during a 5-fold cross-validation. The image quality is assessed using the mean over the fold of the root mean squared error (RMSE) for the magnitude, the mean deviation of the phase, and the structural similarity index measure (SSIM) averaged real and imaginary parts. Fold #2, tested on subset #2, was utilized for further evaluation.

| **Fold of** $\mathrm{NN}_{\mathrm{cx}}^{\mathrm{sag}}$ | **Tested on** | **RMSE of Magnitude**  mean ± std (%) | **Mean Deviation Phase**  mean ± std (°) | **SSIM for Complex**  mean ± std (%) |
| --- | --- | --- | --- | --- |
| #1 | Subset #1 | 3.029 ± 2.052 | 7.228 ± 3.034 | 89.694 ± 4.070 |
| **#2** | **Subset #2** | **1.822 ± 0.694** | **4.245 ± 1.616** | **93.278 ± 2.620** |
| #3 | Subset #3 | 2.312 ± 0.751 | 6.652 ± 2.626 | 90.338 ± 3.690 |
| #4 | Subset #4 | 2.409 ± 0.554 | 6.217 ± 0.819 | 90.029 ± 1.512 |
| #5 | Subset #5 | 2.728 ± 1.979 | 5.466 ± 2.811 | 92.109 ± 3.451 |

Table S3: Predicted image quality of $\mathrm{NN}_{\mathrm{cx}}^{\mathrm{cor}}$ trained and tested on coronal slices during a 5-fold cross-validation. The image quality is assessed using the mean over the fold of the root mean squared error (RMSE) for the magnitude, the mean deviation of the phase, and the structural similarity index measure (SSIM) averaged real and imaginary parts. Fold #2, tested on subset #2, was utilized for further evaluation

| **Fold of** $\mathrm{NN}_{\mathrm{cx}}^{\mathrm{cor}}$ | **Tested on** | **RMSE of Magnitude**  mean ± std (%) | **Mean Deviation Phase**  mean ± std (°) | **SSIM for Complex**  mean ± std (%) |
| --- | --- | --- | --- | --- |
| #1 | Subset #1 | 1.738 ± 0.696 | 3.405 ± 0.991 | 94.604 ± 1.670 |
| **#2** | **Subset #2** | **1.331 ± 0.273** | **2.683 ± 0.600** | **95.598 ± 1.066** |
| #3 | Subset #3 | 1.901 ± 1.560 | 4.028 ± 2.320 | 94.332 ± 2.525 |
| #4 | Subset #4 | 2.137 ± 1.735 | 4.254 ± 1.651 | 93.296 ± 2.566 |
| #5 | Subset #5 | 1.608 ± 0.491 | 2.940 ± 1.098 | 95.145 ± 1.820 |

Table S4: Predicted image quality of $\mathrm{NN}_{\mathrm{cx}}^{\mathrm{all}}$ trained and tested on all slice orientations during a 5-fold cross-validation. The image quality is assessed using the mean over the fold of the root mean squared error (RMSE) for the magnitude, the mean deviation of the phase, and the structural similarity index measure (SSIM) averaged real and imaginary parts. Fold #2, tested on subset #2, was utilized for further evaluation.

| **Fold of** $\mathrm{NN}_{\mathrm{cx}}^{\mathrm{all}}$ | **Tested on** | **RMSE of Magnitude**  mean ± std (%) | **Mean Deviation Phase**  mean ± std (%) | **SSIM for Complex**  mean ± std (%) |
| --- | --- | --- | --- | --- |
| #1 | Subset #1 | 2.325 ± 1.413 | 5.414 ± 2.590 | 91.882 ± 3.664 |
| **#2** | **Subset #2** | **1.907 ± 1.696** | **4.431 ± 2.341** | **92.947 ± 3.735** |
| #3 | Subset #3 | 2.322 ± 1.252 | 6.205 ± 3.455 | 90.795 ± 5.234 |
| #4 | Subset #4 | 2.332 ± 1.157 | 6.065 ± 2.359 | 90.544 ± 3.744 |
| #5 | Subset #5 | 2.323 ± 1.338 | 5.232 ± 2.910 | 91.829 ± 4.553 |

## Supporting Information

### Supporting Information S1: MR imaging and data processing

The study comprising of 15 healthy subjects (age: (40±16) years, max/min: 69/27 years; body mass index (BMI): (25.10±5.37) kg/m^2^, max/min: 37/19 kg/m^2^, skewness: 0.87; male/female: 10/5) was approved by a local ethics committee, and informed consent was obtained for all cases. Data was acquired on a 7T whole-body MRI scanner (Magnetom 7T, Siemens, Erlangen, Germany) utilizing a custom-built 8Tx/8Rx-channel transceiver head coil array (1) optimized for a high transmission efficiency for spectroscopy consisting of eight rectangular loop elements in an elliptical arrangement. The subjects were scanned supine with the forehead positioned in the isocenter.

A data library containing multi-slice, Rx-channel-wise localizer images and multi-slice, Tx-channel-wise B_1_^+^-maps is considered (c.f. Figure S3). In general, for the input of the NNs, a small FA, multi-slice whole-brain gradient echo (GRE) localizer image with all Tx-channels transmitting in a CP+-mode for different slice orientations (transversal/sagittal/coronal) is acquired. The following parameters are used: nominal FA=10°, reference voltage (1Tx)=80 V, TE=2.9 ms, TR=100 ms, TA=1.63-2.43 min, FOV=(256x192) mm^2^, resolution=(2x2) mm^2^, slice thickness=4 mm, 100% distance factor, 15/15/20 interleaved transversal/sagittal/coronal slices, fully sampled k-space, i.e. no parallel imaging or partial Fourier applied. The process is repeated eight times with identical sequence parameters but only one active Tx-channel, shifting the active channel across the scans. Adopting the hybrid approach of Van de Moortele et al. (2), the Tx-channel-wise, orientation-dependent GRE measurements are merged with a 3D actual flip imaging (AFI) dataset obtained for all Tx-coils active (nominal FA=60°, reference voltage (1Tx)=80 V, TR1/TR2=25/125 ms, TE=1.9 ms, TA=9.5 min, FOV=(256x192x256) mm^3^, resolution=(2x2x4) mm^3^, slice thickness=4 mm, 64 slices, fully sampled k-space). Depending on the orientation of the GRE images, the resulting absolute, Tx-channel-wise, multi-slice B_1_^+^-maps are used as an unbiased B_1_^+^-reference (3) serving as a ground truth (GT) for training the NNs with different slice orientations ($\mathrm{NN}_{\mathrm{cx}}^{\mathrm{tra}}$ to $\mathrm{NN}_{\mathrm{cx}}^{\mathrm{all}}$). The complex B_1_^+^-distributions given in degrees are normalized by a fixed value of 60°, reflecting the chosen target FA. The complex-valued localizers are scaled with a corresponding fixed value, ensuring a comparable dynamic range for all datasets. The B_1_^+^-phase is calculated relative to the phase of the superimposed complex-valued B_1_^+^-maps of all Tx-channels (c.f. (4)), i.e., a CP+-based phase, thus resulting in a zero-phase of the summed-up B_1_^+^-data. This approach achieves a smoother data distribution compared to only a single Tx-channel as a phase reference.

From the 15/15/20 transversal/sagittal/coronal slices that covered a larger region than the brain, only 8-11 transversal, 11-13 sagittal, and 14-16 coronal slices are included in the library per subject, depending on the subject's head shape. Binary masks, determined by setting a threshold value for the sum-of-magnitudes of the B_1_^+^-maps, are applied pixel-wise to both input and output data to suppress noise signals outside the brain. For the subsequent pulse design, a manually selected binary mask based on the SOM is chosen as the region of interest covering the brain tissue.

Data reconstruction, the definition of the binary masks, and the application of the hybrid approach are performed on a separate workstation (12 cores with 2.1 GHz, 128 GB RAM) using a custom-built toolbox written in Matlab (Version 2020a, The Mathworks, Natick, MA, USA).

### Supporting Information S2: Pulse design

Subject-specific static (4,5) and dynamic pTx pulses (6) based on the predicted (PR) whole-brain B_1_^+^-maps are designed to investigate how the accuracy of the PR B_1_^+^-maps impacts the FA homogeneity when applying pTx. Both static B_1_^+^-shimming and dynamic 3D excitation are optimized on the PR and GT B_1_^+^-maps. For static B_1_^+^-phase-only shimming (3,4,7), the individual 2D B_1_^+^-slices are considered independently, and the Tx-phases were optimized to determine the B_1_^+^-shim vector **b**_Hom_. The B_1_^+^-shimming results applying **b**_Hom_ to the GT are compared to the shim setting **b**_CP+_ for the GT when all Tx-channels transmit in CP+-mode. Furthermore, dynamic 4 kt-points pTx pulses are designed using the small FA spatial domain method (5–7). The excitation profile is optimized for the 3D volume of each subject's brain, considering axial slices. Bloch simulations investigate the accuracy using both the GT maps. As a metric, the coefficient of variation (CV) is used as a surrogate for spatial homogeneity.

### Supporting Information S3: Experimental application

High-resolution GRE images are acquired in two in-vivo test subjects (age: 28/35 years; body mass index (BMI): 23.6/18.14 kg/m^2^; 1male/1female) to validate the approach's feasibility in a subject-specific calibration pipeline. For the B_1_^+^-prediction, one separate NN is trained on data from all slice directions and all 15 subjects. The 4 kT-point pulses used to acquire the high-resolution GRE images are calculated as described in Supporting Information S2 on the PR and GT.

### Supporting Information S4: B_1_^+^-maps and image quality

Figure S5 depicts axial PR B_1_^+^-maps compared to reference as the GT for the magnitude and phase of the complex, Tx-channel-wise summed-up data in eleven slices of unseen test subject #1. The best prediction quality regarding the SSIM for complex numbers (highlighted in blue) was found for anatomically superior slices. In contrast, the worst performance was seen for inferior slices (marked in green). An example slice located in the middle was selected as well. The combined PR qualitatively matches the GT data in magnitude and phase distributions across all slices. The magnitude difference $\Delta B_{1}^{+}$ shows larger differences in more central regions with higher absolute magnitude. $\mathrm{NN}_{\mathrm{cx}}^{\mathrm{tra}}$ yields a prediction of the summed-up B_1_^+^-maps with a mean relative error for the magnitude of (0.94±0.29)% and a mean absolute difference in the phase of (1.90°±0.68°) in the brain tissue. The results of the combined B_1_^+^-maps for test subjects #2 and #3 predicted by $\mathrm{NN}_{\mathrm{cx}}^{\mathrm{tra}}$, as well as the results for $\mathrm{NN}_{\mathrm{cx}}^{\mathrm{sag}}$and $\mathrm{NN}_{\mathrm{cx}}^{\mathrm{cor}}$ for subject #1, trained and tested on sagittal and coronal slices, can be found in Figure S6 and Figure S7.

Figures S8A and S8D show the B_1_^+^-magnitude and phase maps for the channel-wise PR of $\mathrm{NN}_{\mathrm{cx}}^{\mathrm{tra}}$ compared to the B_1_^+^-reference as the GT for the selected inferior slice of subject #1 (marked in green). As for the channel-combined case shown in Figure S5, the channel-wise PR data matches the GT data, not only in magnitude but particularly in the phase distributions. The phase only shows differences in regions of very low magnitudes, c.f. Tx-channels 1, 5, and 6. Only minor deviations are visible at high magnitude levels in the absolute error ΔB_1_^+^ between PR and GT. Vertical and horizontal profiles through the channel-wise magnitude maxima for the DL-based (black) and GT B_1_^+^-maps (red) are provided in Figure S8 B/C/E/F for all Tx-channels. Overall, the PR qualitatively matches the GT in the human brain for both magnitude and phase. Residual deviations are evident around the maximum for the magnitude and at wraps or singularities for the phase distribution. Furthermore, Figure S9 compares channel-wise B_1_^+^-data of the exemplary slices for all three unseen subjects.

Figure S10 examines the pixel-wise correlation between the PR B_1_^+^-maps and the reference for the brain tissue, considering magnitude and phase distributions for all Tx-channels. A linear correlation between PR and GT becomes evident, leading to mean Pearson coefficients of ρ = (0.966±0.018) for the magnitude and ρ = (0.884±0.142) for the phase. Tx-channels 1, 2, 6, and 7 exhibit the most significant deviation from linear behavior, resulting in the lowest Pearson coefficients among the cases presented.

Figure S11 also depicts the combined B_1_^+^-maps for one unseen subject of Fold #3, which yielded the lowest performance during the 5-fold cross-validation.

### Supporting Information S5: Effect of B_1_^+^-quality on RF pulse design for static pTx

Figure S12A shows the results for phase-only static pTx in the example slice, the best and worst case of unseen subject #1 when considering the shim setting **b**_CP+_ for transmission in CP+-mode and the homogeneity-optimized shim **b**_Hom_.on the PR and GT applied to the reference data B_1_^+^_GT_. Optimizing the Tx-phase by considering the CV as a cost function improves homogeneity in the selected ROI at the expense of a lower magnitude level. A quantitative evaluation regarding the CV in the brain for static pulse design is given in Figure S12B by comparing the application of the homogenous shim **b**_Hom_ calculated on the reference B_1_^+^_GT_, as well as on the predictions B_1_^+^_PR_ of $\mathrm{NN}_{\mathrm{cx}}^{\mathrm{tra}}$ and $\mathrm{NN}_{\mathrm{real}}^{tra,1}$ to $\mathrm{NN}_{\mathrm{real}}^{tra,3}$ to the GT. The mean CV of the GT decreases from 21.9% (range=10.2%-30.8%) to a value of 11.1% (range=6.1%-18.5%) when relying on **b**_Hom_ calculated on the GT. Applying **b**_Hom_ calculated on the PR of $\mathrm{NN}_{\mathrm{cx}}^{\mathrm{tra}}$ to the GT leads to generally higher CV values, leading to the best mean CV values around 15.6% (range=8.2%-24.4%).

1. Seifert F, Pfeiffer H, Mekle R, Waxmann P, Ittermann B. 7T 8-Channel PTx Head Coil with High B1+ Efficiency Optimized for MRS. In: Proceedings of the 24th Annual Meet. ISMRM, Singapur, Singapur: Abstract 3545; 2016.

2. Van de Moortele PF, Snyder C, DelaBarre L, Adriany G, Vaughan T, Ugurbil K. Calibration Tools for RF Shim at Very High Field with Multiple Element RF Coils: from Ultra Fast Local Relative Phase to Absolute Magnitude B1+ Mapping. In: Proceedings of the 15th Annual Meeting of ISMRM, Berlin, Germany: Abstract 1676; 2007.

3. Dietrich S, Aigner CS, Kolbitsch C, Mayer J, Ludwig J, Schmidt S et al. 3D Free-breathing multichannel absolute B1+ Mapping in the human body at 7T. Magn. Reson. Med. 2021;85:2552–2567.

4. Krüger F, Aigner CS, Hammernik K, Dietrich S, Lutz M, Schulz-Menger J et al. Rapid estimation of 2D relative B1+-maps from localizers in the human heart at 7T using deep learning. Magn. Reson. Med. 2022;89:1002–1015.

5. Cao Z, Yan X, Grissom WA. Array-compressed parallel transmit pulse design. Magn. Reson. Med. 2016;76:1158–1169.

6. Grissom WA, Khalighi MM, Sacolick LI, Rutt BK, Vogel MW. Small-tip-angle spokes pulse design using interleaved greedy and local optimization methods. Magn. Reson. Med. 2012;68:1553–1562.

7. Aigner CS, Dietrich S, Schmitter S. Three-dimensional static and dynamic parallel transmission of the human heart at 7 T. NMR Biomed. 2021;34:1–15.
